# Synthesis, characterization and DNA interaction studies of new triptycene derivatives

**DOI:** 10.3762/bjoc.10.130

**Published:** 2014-06-05

**Authors:** Sourav Chakraborty, Snehasish Mondal, Rina Kumari, Sourav Bhowmick, Prolay Das, Neeladri Das

**Affiliations:** 1 Department of Chemistry, Indian Institute of Technology Patna, Patna 800 013, Bihar, India

**Keywords:** abasic site, DNA damage, ethynyl, pUC19 plasmid, triptycene tripod

## Abstract

A facile and efficient synthesis of a new series of triptycene-based tripods is being reported. Using 2,6,14- or 2,7,14-triaminotriptycenes as synthons, the corresponding triazidotriptycenes were prepared in high yield. Additionally, we report the transformation of 2,6,14- or 2,7,14-triaminotriptycenes to the corresponding ethynyl-substituted triptycenes via their tribromo derivatives. Subsequently, derivatization of ethynyl-substituted triptycenes was studied to yield the respective propiolic acid and ethynylphosphine derivatives. Characterization of the newly functionalized triptycene derivatives and their regioisomers were carried out using FTIR and multinuclear NMR spectroscopy, mass spectrometry, and elemental analyses techniques. The study of the interaction of these trisubstituted triptycenes with various forms of DNA revealed interesting dependency on the functional groups of the triptycene core to initiate damage or conformational changes in DNA.

## Introduction

Triptycene is the simplest member of the class of compounds called iptycenes. Structurally, iptycenes have a number of arene rings joined together to form the bridges of [2.2.2]bicyclic ring systems. In triptycene, the bicyclic ring is decorated with three benzene rings in a paddlewheel configuration. Although, Barlett reported the first synthesis of triptycene in 1942 [1], research in the development of supramolecular systems based on triptycene is still in its early stages. Design and synthesis of triptycene derivatives has gained research attention since the beginning of this century [2–6]. In general, triptycene derivatives have a high level of symmetry associated with a very rigid framework. Owing to this property, triptycene derivatives have found application as building blocks in the design of synthetic molecular machines, polymers, supramolecules and other smart materials [2–8]. In order to explore more applications of triptycenes, there is a need to design newer derivatives of triptycene having specific functional groups.

It is well established that aromatic azides are an important class of compounds having various interesting applications in organic chemistry [9–11] as well as material science [12–15] and drug discovery [16–18]. Organic azides in general are popular in recent times because of their use as synthons in Cu(I)-catalyzed “click chemistry” [19]. It is also well known that organic molecules having terminal ethynyl functional groups are very useful synthons for the design of interesting materials in polymer and supramolecular chemistry [20–25].

Herein we report, the syntheses of 2,6,14- and 2,7,14-triazido and 2,6,14- and 2,7,14-triethynyl substituted triptycenes. We also describe the syntheses of 2,6,14- and 2,7,14-triptycenetripropiolic acid and tris(ethynyldiphenylphosphino)triptycene derivatives via functional group transformation of triethynyltriptycenes. All the eight trisubstituted triptycene derivatives have been fully characterized by FTIR and multinuclear NMR spectroscopy, mass spectrometry, and elemental analyses techniques. We have also studied the photophysical properties of all triptycene derivatives.

Furthermore, we have explored a new face of triptycene-based molecules, viz., interaction of tripodal triptycene-based molecules (with various functionalities) with DNA. According to a literature survey, many metal-free small molecules are known to modify structural organization of DNA through recognition, binding, cleavage or crosslinking and reportedly have widespread applications in biology [26]. Targeting of DNA by several DNA-damaging agents have been studied with numerous compounds with the objective to treat cancer cells [27]. However, side effects including the risk of secondary cancers have been witnessed and the current trend encourages introduction of inhibitors of enzymes whose essential substrate is DNA [28]. Recent reports have shown that selected triptycene derivatives show anticancer activities by virtue of inhibition of topoisomerase [29–30], obstruction of macromolecule synthesis [31], blocking of nucleoside transport [32], induction of caspase activities [33] and poly(ADP)-ribose polymerase-1 (PARP) cleavage [34], and at the same time does not inflict any significant damage to the DNA structure even when present as a ligand in organometallic complexes of palladium [35]. We believe triptycene could be one such compound, which can act against several metabolic pathways and yet be non-DNA damaging in nature. However, to the best of our knowledge, till date there are only a few reports in literature about interaction of triptycene derivatives with DNA and hence research in this area is presumably in the nascent stage. Our studies indicate that trisubstituted triptycenes interact with DNA and the extent of these interactions depends on the functionality of the substituents. However, there is no marked difference in the extent of interaction with DNA between a given pair of regioisomers.

## Results and Discussion

**Scheme 1:**
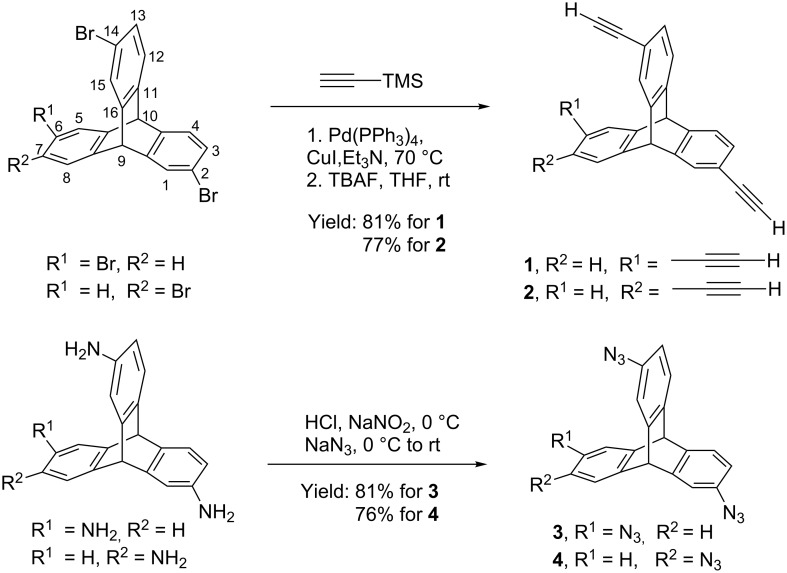
Synthesis of TETs **1** and **2** and TATs **3** and **4**.

**Synthesis and characterization of ethynyl- and azide-substituted triptycenes**. Using appropriate tribromotriptycenes as synthons [36], syntheses of triethynyltriptycenes (TETs) **1** and **2** were efficiently realized in two steps (Scheme 1). In the presence of Pd(0) catalyst and cuprous iodide (cocatalyst), Sonogashira cross-coupling reaction of 2,6,14-tribromotriptycene with 3.0 equiv of (trimethylsilyl)acetylene in triethylamine, followed by deprotection with TBAF in THF at room temperature yielded 2,6,14-triethynyltriptycene (**1**, yield = 81%). Using similar reaction conditions, 2,7,14-triethynyltriptycene (**2**) was obtained from the corresponding tribromide in 77% isolated yield.

For the syntheses of triazidotriptycenes (TATs) **3** and **4**, our strategy was to use the well established chemistry of conversion of aniline to the aryl diazonium ion and subsequent reaction with azide nucleophile. As shown in Scheme 1, treatment of the appropriate triaminotriptycene [36] with an aqueous solution of sodium nitrite in presence of hydrochloric acid and subsequent reaction with sodium azide yielded the triazidotriptycene **3** and **4** in 81% and 76% yield respectively.

The newly synthesized triethynyltriptycenes (TETs) and triazidotriptycenes (TATs) were characterized by FTIR, multinuclear NMR (^1^H and ^13^C), LRMS, and elemental analyses. In FTIR spectra a strong band at 3266 cm^−1^ (ν C≡C–H str.) and a medium band at 2101 cm^−1^ (ν C≡C str.) indicated formation of **1**. Similarly, bands at 3261 cm^–1^ (s, ν C≡C–H str.) and 2105 cm^–1^ (m, ν C≡C str.) in FTIR spectra suggested formation of **2**. In ^1^H NMR spectra of **1** and **2**, two singlet peaks between 5.27–5.40 ppm were assigned to the bridgehead protons of triptycene moiety. The ethynyl protons of **1** and **2** appear as singlet at 2.90 ppm and 2.97 ppm respectively. As expected, in the ^13^C NMR spectra, aromatic carbons show twelve and six peaks for **1** and **2** respectively. Two peaks between 84.0 to 77.0 ppm in ^13^C NMR spectra correspond to ethynyl carbons of **1** and **2** (Supporting Information File 1).

The FTIR spectrum of **3** exhibits a strong absorption band at 2108 cm^–1^ (ν N=N=N str.). Similarly, a band at 2113 cm^–1^ (s, ν N=N=N str.) suggests the formation of **4**. In the proton NMR spectra of the azide derivatives **3** and **4**, peaks corresponding to the aromatic and bridge head protons of the central triptycene core show similar splitting modes but different chemical shifts when compared with that of the corresponding triaminotriptycene derivative. In addition, compounds **3** and **4** were characterized by ^13^C NMR, LRMS and elemental analysis (Supporting Information File 1).

**Functional group transformation of ethynyl-substituted triptycenes**. Molecules with multiple carboxylate functional groups are an important class of compounds that have often been used as building blocks in the design of metal-organic frameworks (MOFs) with applications including but not limited to catalysis and gas uptake/storage [37–42]. It is well known that terminal alkynes can be conveniently subjected to carboxylation using CO_2_ to yield the corresponding alkynyl carboxylic acids.

**Scheme 2:**
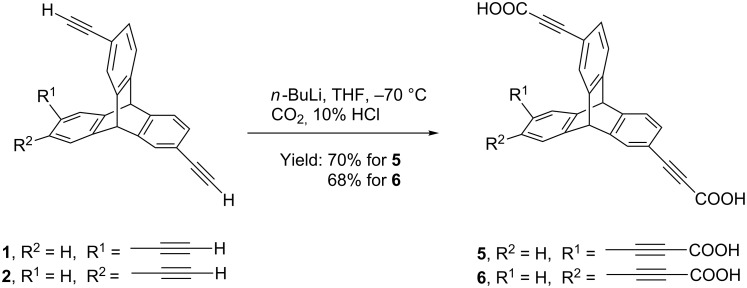
Synthesis of triptycenetripropiolic acid (TPA) **5** and **6**.

As shown in Scheme 2, 2,6,14-triptycenetripropiolic acid (TPA) **5** was efficiently synthesized by the treatment of 2,6,14-triethynyltriptycene with *n*-BuLi at –70 °C, followed by bubbling of CO_2_ gas through the reaction mixture. The lithiated tricarboxylate thus obtained was treated with dilute hydrochloric acid to obtain the desired 2,6,14-triptycenetripropiolic acid as sole isolated product in high yield (70%). Using similar reaction conditions, 2,7,14-triptycenetripropiolic acid (**6**) was obtained in 68% yield.

In FTIR spectrum of **5**, bands at 1700 cm^−1^ (s, ν C=O str.) and 2206 cm^−1^ (w, ν C≡C str.) indicate the formation of the desired propiolic acid. Similarly in case of **6**, the corresponding bands appeared at 1710 cm^−1^ (s, ν C=O str.) and 2210 cm^−1^ (w, ν C≡C str.). In the proton NMR spectra of **5** and **6**, peaks corresponding to aromatic and bridge head protons appear in the range 7.32–7.80 ppm and 5.85–6.00 ppm respectively. As expected, ^13^C{^1^H}NMR spectra of triptycene tricarboxylic acids **5** and **6** have additional peaks (153–155 ppm) in comparison to that for the corresponding triethynyltriptycene derivatives due to the carbonyl carbon of the carboxylic acid functional group (Supporting Information File 1).

**Scheme 3:**
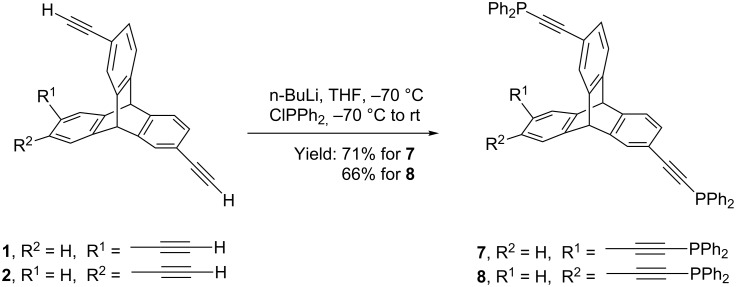
Synthesis of triphosphinotriptycenes (TPT) **7** and **8**.

Additionally, we also report two isomers of tris(ethynyldiphenylphosphino)triptycene (TPT) **7** and **8** that were synthesized by functional group transformation of ethynyl-substituted triptycenes (Scheme 3). Reaction of appropriate TETs with *n*-BuLi at −70 °C followed by reaction with chlorodiphenylphosphine yielded the corresponding TPTs **7** and **8** in 71% and 66% yield, respectively. In the ^1^H NMR spectrum of the product, absence of the peak corresponding to terminal ethynyl proton and presence of multiplet peaks in the aromatic region (7.30–7.39 ppm and 7.59–7.65 ppm) suggested formation of the desired triphosphine. A single peak in the ^31^P NMR spectrum of the product **7** and **8** between 8.9–9.1 ppm confirms the formation of triphosphines.

### Biological studies: Interaction of triptycene derivatives with DNA

**Figure 1:**
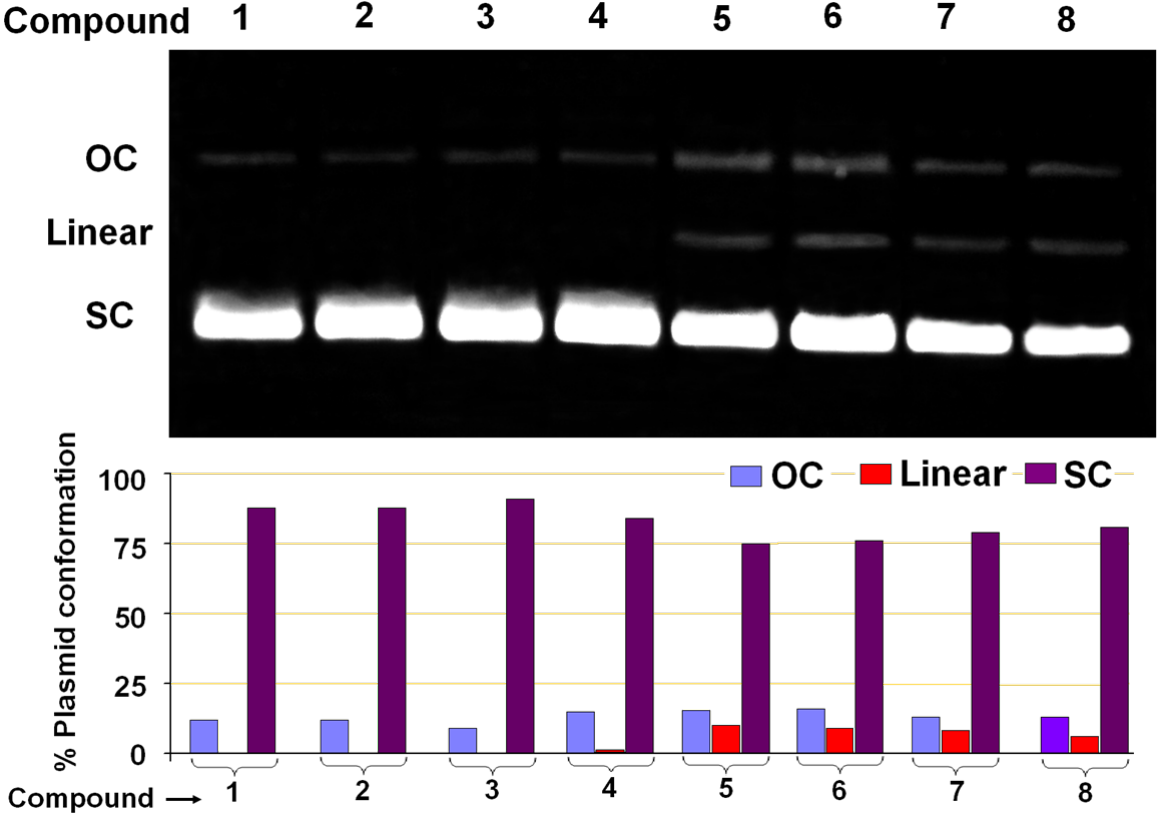
Nuclease activities of the triptycene derivatives **1–8**. Agarose gel (top) shows results of the incubation of pUC19 plasmid DNA with triptycene derivatives and the bar diagram (bottom) represents the quantitative analysis of the gel bands. This shows the amount of different forms of plasmid present after incubation with the compounds. OC and SC represent the open circular and the supercoiled form of the plasmid, respectively.

All the triptycene derivatives, when incubated with plasmid DNA show different degree of nuclease activity on supercoiled pUC19 plasmid DNA. The nuclease activity of the trisubstituted triptycene derivatives TETs **1** and **2** and TATs **3** and **4** is minimal and the incubation of plasmid with those compounds for 24 hours only results in the generation of a small amount of the open circular (OC) or the nicked form without the formation of any linear form (Figure 1). However, both the linear as well as the OC form of the plasmid were observed following incubation with the TPAs **5** and **6** and TPTs **7** and **8**. Comparison between the TPAs and TPTs indicates that the former have slightly higher nuclease activity. Complexes with triphenylphosphine as ligands have been shown to bind to DNA in a non-intercalative mode due to the waggling of the phenyl rings [43]. A similar effect might be operational also in the case of the TPTs **7** and **8**, which prevents deep penetration of the whole compound into the core of the phosphate backbone. Thus, any nuclease activity of these compounds is attributed to the interaction of the electronic states of the compounds with significant contribution from the phosphorus atom with the phosphate backbone of DNA from just sitting on the grooves. The cleavage of supercoiled plasmid by the TPAs (**5** and **6**) indicates possible formation of hydrogen bonds between the compounds and the minor grooves of the DNA, in addition to the electrostatic forces that destabilizes the DNA double helix and the bases, finally resulting in hydrolysis of the phosphate backbone [44–45]. We conducted DNA melting temperature (*T*_m_) studies and observed that the *T*_m_ of ctDNA decreased by ~3.5– 4 °C when incubated with the TPAs **5** and **6** (see Supporting Information File 1, Figure S1). The decrease in *T*_m_ at physiological pH for **5** and **6** clearly points out to the local structural distortion brought about by the compounds that can initiate the hydrolytic cleavage of the DNA [46]. Such an effect of significant change in *T*_m_ was not observed for compounds **1**–**4** and **7** and **8**.

**Figure 2:**
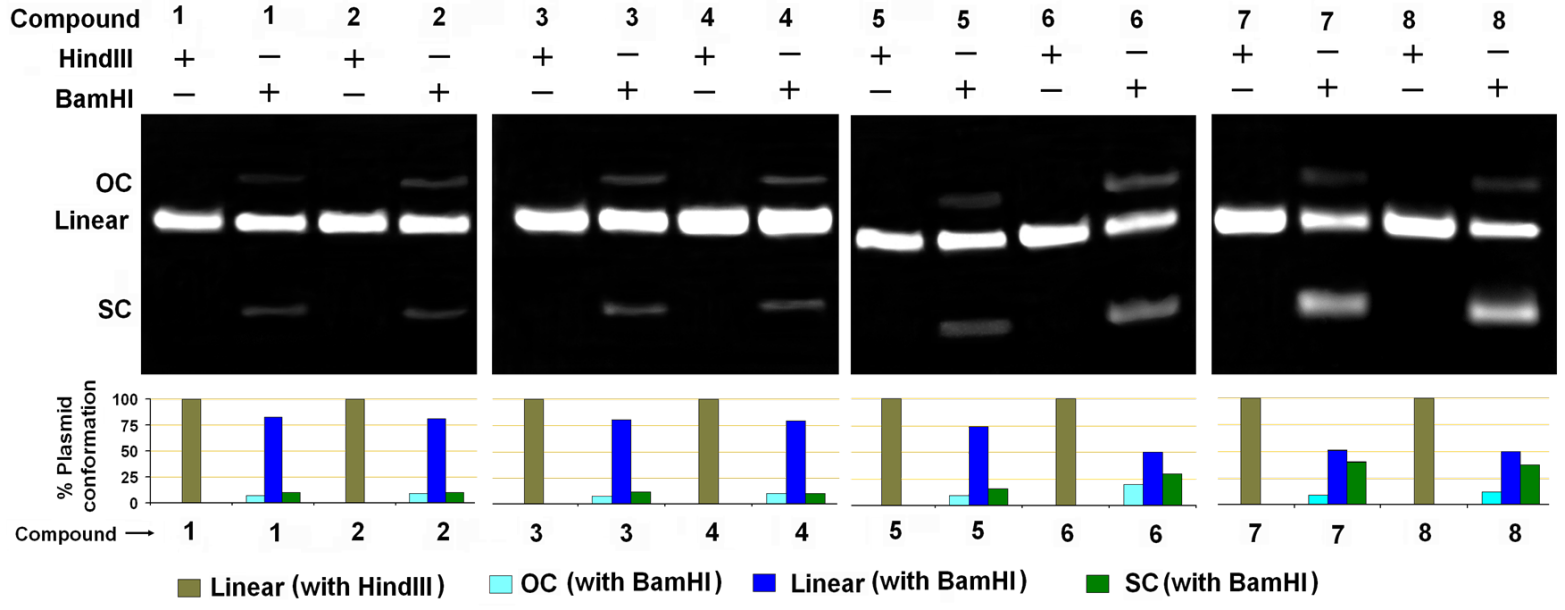
Effect of triptycene derivatives on the restriction endonuclease activity of HindIII and BamHI enzymes on pUC19 plasmid. Agarose gel (top) shows the presence of the different forms of the plasmid following incubation with the compound and the respective restriction endonuclease. Quantitative estimation of the different forms of pUC19 in the corresponding gel lanes are shown in the bar diagram (bottom). OC and SC represent the open circular and the supercoiled form of pUC19.

**Base selectivity**: Restriction endonucleases recognise particular bases in the DNA and subsequently cleave the DNA by hydrolysis of the phosphodiester bonds. Inhibition of endonuclease activity in presence of any compound signifies that the compound interacts with the cleavage site of the enzyme [47]. We used *Hind*III (recognition site A↓AGCTT) and *BamH*I (recognition site G↓GATCC) restriction endonucleases to evaluate the affinity of triptycene derivatives toward G/C or A/T base sequence of DNA. While the compounds (200 µM) were unable to inhibit the endonuclease activity of *Hind*III, inhibition of the action of *BamH*I under similar experimental conditions was exhibited by all the triptycene derivatives being reported herein (Figure 2). This suggests that all the compounds have more affinity towards G/C than compared to A/T bases in DNA. Interestingly, the highest amount of inhibition of *BamH*I was recorded for the TPTs **7** and **8**, followed by the TPAs **5** and **6**, all of which have displayed appreciable nuclease activity on plasmid DNA.

**Figure 3:**
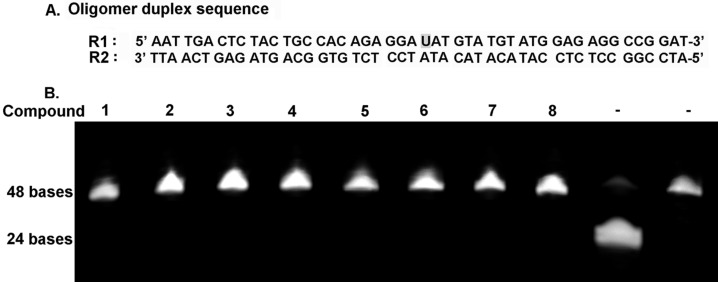
(A) Oligomer duplex sequence that was used to study the effect of triptycene derivatives on the abasic site in DNA. The site of abasic site generation is denoted as U (Uracil). (B) Effect of the triptycene derivatives on abasic sites revealed by polyacrylamide gel electrophoresis (PAGE). The last two gel lanes do not contain any compounds and oligomer duplex DNA in those lanes were treated with UDG + ApeI and UDG only, respectively.

**Cleavage of abasic sites:** Abasic sites in the DNA duplex are generated when a purine (A/G) or a pyrimidine (C/T) base is stripped off from the DNA strand and this is considered as the most common type of DNA damage lesions. We generated one abasic site in a 48-base pair long oligomer duplex by treating the DNA (Figure 3) with Uracil DNA Glycosylase (UDG) enzyme, which excises one uracil residue present in the middle of the oligomer duplex (Figure 3). The oligomer duplexes after UDG treatment were incubated with 200 µM of the compound (**1–8**) for 24 hours in 10 µL reaction mixture. We found that none of the compounds were able to generate single strand breaks at the abasic site by hydrolysis of the phosphate backbone (Figure 3). This signifies that though few compounds are able to hydrolyse phosphodiester bonds in plasmids, none of the compounds are able to reproduce that act, when a base is missing from the DNA duplex. Thus the interaction of the compounds is mainly with the DNA bases that subsequently contribute to the hydrolytic activity of the compounds if any.

**Spectroscopic studies:** The mode of binding of compounds to calf thymus DNA (ctDNA) was evaluated by an ethidium bromide (EtBr) displacement assay [48]. Binding of a compound to DNA results in the displacement of the intercalated EtBr, and subsequently quenches the fluorescence of the EtBr–DNA complex by releasing EtBr in the solution. The fluorescence intensity of the EtBr–DNA complex did not decrease appreciably after addition of the compounds (**1–8**, 200 μM). However, the TPAs **5** and **6** showed some displacement of the intercalated EtBr, as evident from the steady-state fluorescence data (Supporting Information File 1, Figure S2). This indicates that the mode of interaction of the compounds with DNA may not be intercalation except for the triptycene derivatives **5** and **6**.

**Figure 4:**
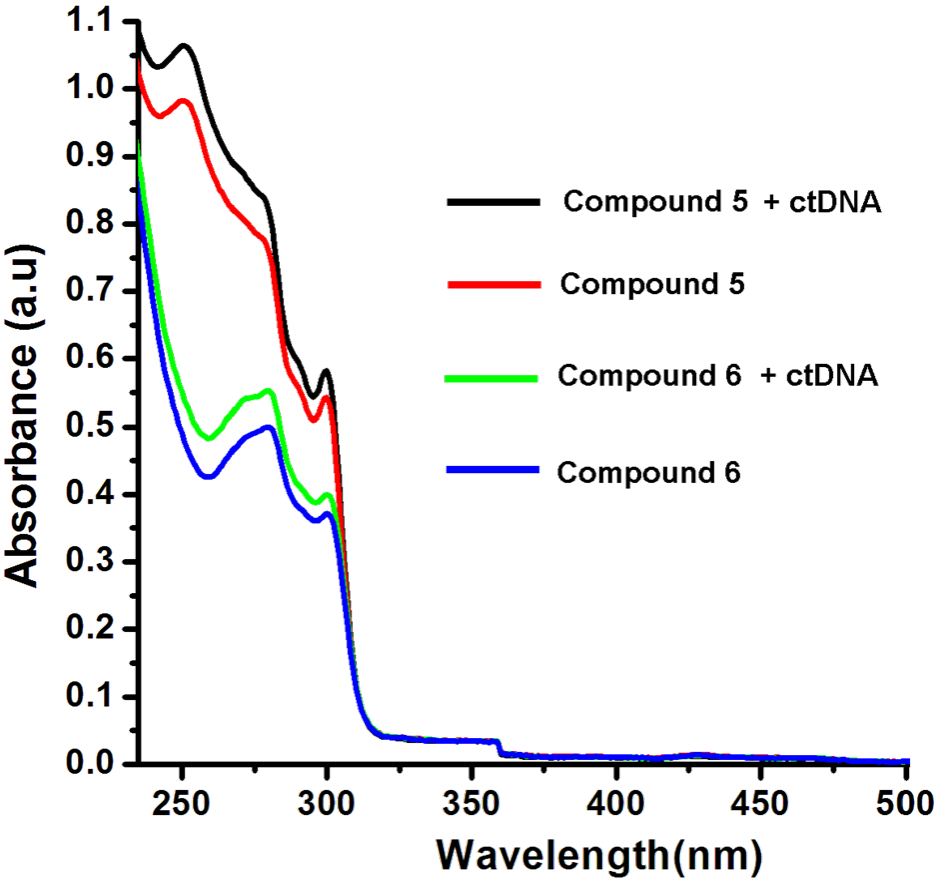
UV–vis spectra showing the hyperchromic effect after addition of ctDNA (20 μM) to **5** and **6** (80 μM).

No noticeable changes in the wavelength as well as absorption maxima were observed in the UV–visible absorption spectra of the compounds **1**–**4** and **7**, **8** in presence of ctDNA (Supporting Information File 1, Figure S3–S6). However, a slight hyperchromic shift in the absorption peak of the TPAs **5** and **6** was observed after addition of ctDNA (Figure 4). The observed hyperchromic effect due to the addition of DNA to **5** and **6** is indicative of the electrostatic interaction or partial destabilization of the helix structure of DNA [49–50].

### Photophysical characterization

**Figure 5:**
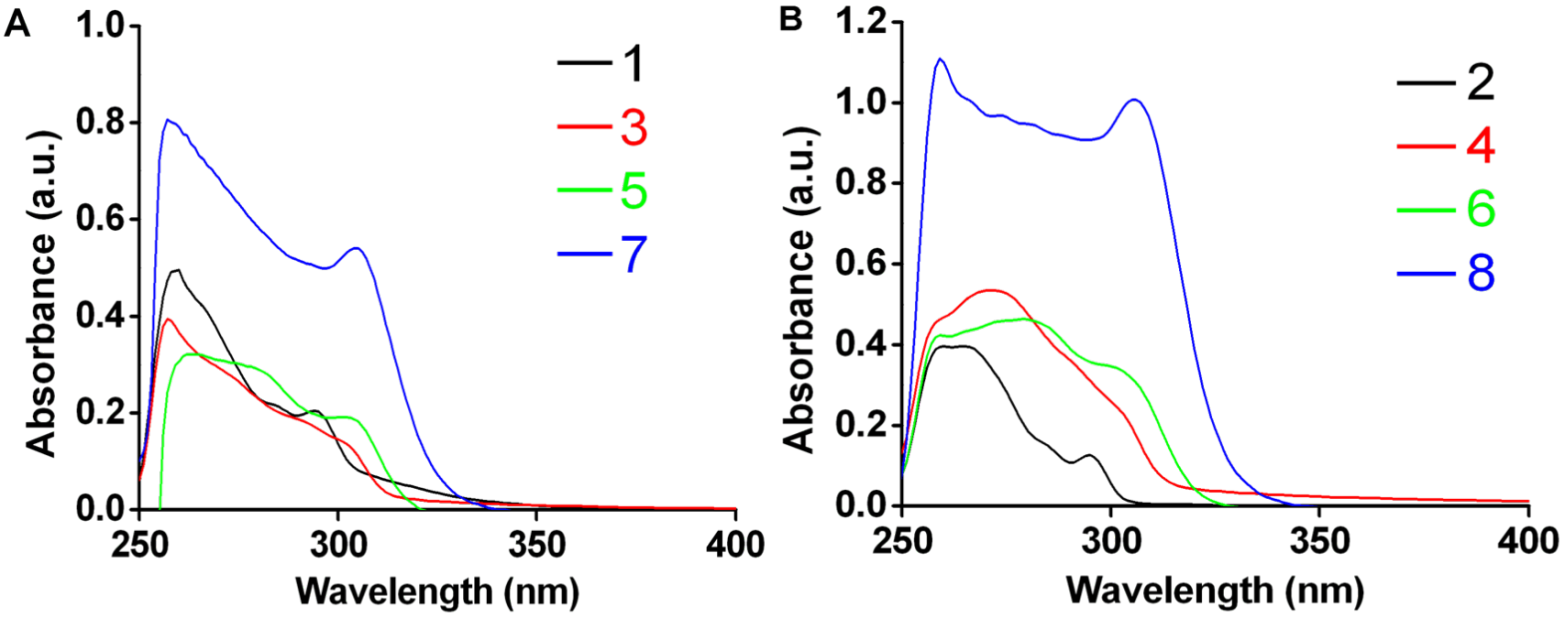
UV–vis absorption spectra of (A) 2,6,14-trisubstituted triptycene derivatives **1**, **3**, **5** and **7** in DMSO (15 μM) at 298 K; (B) 2,7,14-trisubstituted triptycene derivatives **2**, **4**, **6** and **8** in DMSO (15 μM) at 298 K.

**Ground state properties:** The UV−visible absorption spectra of **1–8** are shown in Figure 5. As expected, the electronic spectrum in case of each triptycene derivative exhibits transitions in the UV region only. UV–vis absorbance spectra of the TETs **1** and **2** have absorption bands at 295 nm (ε: 1.35 × 10^4^ M^−1^ cm^−1^ for **1** and 8.53 × 10^3^ M^−1^ cm^−1^ for **2**) and 260 nm (ε: 3.29 × 10^4^ M^−1^ cm^−1^ for **1** and 2.64 × 10^4^ M^−1^ cm^−1^ for **2**) which are attributed to π–π* transitions in TETs. In the absorption spectrum of TAT **3**, the most intense band (due to π–π* transitions) was centered at 257 nm (ε: 2.61 × 10^4^ M^−1^ cm^−1^) whereas in case of the **4,** the same was observed at 270 nm (ε: 3.58 × 10^4^ M^−1^ cm^−1^). The absorption spectra of **3** and **4** have a broad shoulder at around 300 nm, and this is due to n–π* transitions in the azides. Triptycene based tricarboxylic acids **5** and **6** exhibited two broad absorption bands centered at 280 nm (ε: 1.94 × 10^4^ M^−1^ cm^−1^ for **5** and 3.06 × 10^4^ M^−1^ cm^−1^ for **6**) and 300 nm (ε: 1.3 × 10^4^ M^−1^ cm^−1^ for **5** and 2.26 × 10^4^ M^−1^ cm^−1^ for **6**). These bands are due to π–π* (aromatic) and n–π* (carboxylic group) transitions.

In case of the trisphosphines **7** and **8**, two strong absorption bands at 300 nm (ε: 3.6 × 10^4^ M^−1^ cm^−1^ for **7** and 6.7 × 10^4^ M^−1^ cm^−1^ for **8**) and 260 nm (ε: 5.37 × 10^4^ M^−1^ cm^−1^ for **7** and 7.38 × 10^4^ M^−1^ cm^−1^ for **8**) are observed, similar to that in case of the ethynyl derivatives. However, the higher magnitude of the absorbance in the case of the phosphines relative to that of the ethynyl derivatives is due to the large number of phenyl groups present in the former.

**Exited state properties:** The DMSO solutions of the ethynyl derivatives **1** and **2** are found to be weakly fluorescent with an emission maximum at 311 nm upon excitation at 260 nm. The corresponding Stokes shift is ~51 nm (Supporting Information File 1, Figure S7) for both **1** and **2**. The quantum yields (Φ) of pure **1** and **2** were found to be 0.016 and 0.022 respectively (aqueous solution of L-tryptophan was used as the reference, Φ = 0.15).

## Conclusion

In conclusion, synthesis and structural characterization of trisubstituted ethynyl and azide triptycenes have been reported. The facile functional group transformations of triethynyl substituted triptycenes to the triphosphine and tripropiolic acid derivatives, in high yields have also been reported. The various functionalized triptycene derivatives have been characterized by FTIR, multinuclear NMR, mass spectrometry and elemental analyses. The functional group dependent interaction of triptycene derivatives with DNA was evaluated in detail and our studies suggest most of the triptycene derivatives are non-DNA damaging and interact weakly with DNA. However, the tripropiolic acid and triphosphine derivatives showed some nuclease activity on plasmid DNA. The results also indicate that triptycene derivatives have more affinity toward guanine–cytosine bases than adenine–thymine bases of DNA. Further studies are currently underway in our lab and include the syntheses of newer derivatives of triptycene, a study of the mechanism of interaction of triptycene derivatives with DNA in more detail, and the use of triptycene derivatives as building blocks in supramolecular chemistry.

## Experimental

Methods of preparation of compounds **1**–**8** and their characterization data are given in Supporting Information File 1.

### Biological studies

**General:** pUC19 plasmid, Uracil DNA Glycosylase (UDG) enzyme, APE1 enzyme, *BamH*I, and *Hind*III restriction endonucleases were purchased from New England Biolabs (USA). HPLC-purified monouracil-containing synthetic deoxyoligomers were purchased from Sigma-Aldrich and deoxyoligomer concentrations were adjusted as per the manufacturer’s yield report. Calf thymus DNA (ctDNA), ethidium bromide (EtBr), chemicals for buffer preparation and synthesis were purchased from Sigma-Aldrich or Alfa Aesar and were used as received. Sybr-Gold^®^ nucleic acid stain was obtained from Invitrogen. Gel images were captured by syngene G:BOX gel documentation system.

#### Evaluation of nuclease activity and sequence selectivity

pUC19 (250 ng) was incubated with 200 µM of each of the compounds at 37 °C for 24 hours in 20 µL reaction volume in 10 mM sodium phosphate buffer (pH 7.2). The cleavage of pUC19 by the compounds was monitored on 1.25% (w/v) agarose gels run at 100 volts for 1.5 h in 1X TAE buffer and stained with ethidium bromide and images captured and quantified.

In order to find out the sequence selective binding of compounds with DNA, incubation of pUC19 (250 ng) with the compounds (200 µM) were followed by further incubation with 5 units of *Hind*III and *BamH*I separately for 1 hour at 37 °C. The results of incubation were confirmed by agarose gel electrophoresis. Quantitative estimation was done using the following equation.


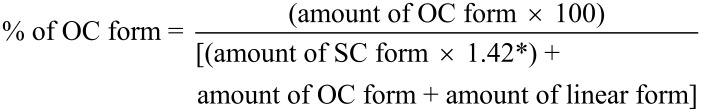


*1.42 = correction factor for supercoiled plasmid.

#### Abasic site cleavage assay

The oligomer duplex (48 base pair) having one deoxyuracil residue in the middle of one of the strands (Figure 3) was treated with 5 units of UDG enzyme in appropriate buffer for 1 h at 37 °C followed by incubation with the compounds (200 µM) at 37 °C for 24 hour in 10 mM sodium phosphate buffer (pH 7.2). The control DNA samples were further incubated with 5 units of APE1 enzyme for 1 hour at 37 °C and analyzed by 25% denaturing PAGE run in tris-taurine-EDTA (TTE) buffer at pH 8.0, stained with Sybr-Gold^®^, gel images acquired and quantified.

#### Fluorescence and absorption spectroscopy

The ethidium bromide (EtBr) displacement assay was carried out with a Horiba Jobin Yuon Floromax-4 spectrofluorometer using a 3 mm pathlength microcuvette. The samples were excited at 540 nm wavelength, and the emission range was set between 550 nm and 800 nm for all fluorescence measurements. The florescence measurements were performed with samples containing 30 µM DNA oligomer duplex, 5 µM EtBr and 200 µM of each of the compounds in 1 mM sodium phosphate buffer (pH 7.2), respectively. The samples along with the controls were incubated at 37 °C for 24 h before the fluorescence measurement. The electronic absorption spectra (UV–visible) of the compounds in presence of the ctDNA (pH 7.2) were recorded on a Shimadzu UV-1601 spectrophotometer. The absorbance of each sample was measured keeping the concentration of the compounds (80 μM) and DNA (20 μM) constant.

## Supporting Information

The Supporting Information provides information about the general synthetic procedure and spectroscopic data for compounds **1**–**8**, ^1^H, ^13^C{^1^H} and ^31^P{^1^H} NMR spectra and LRMS for compounds **1**–**8**, emission and excitation spectra of **1** and **2** and UV–vis and fluorescence spectroscopic data showing the interaction of ctDNA with derivatives **1**–**8**, DNA melting temperature curve with **5** and **6**.

File 1 Synthesis and characterization of compounds **1**–**8**.

## References

[1] Bartlett P D, Ryan M J, Cohen S G (1942). J Am Chem Soc.

[2] Chong J H, MacLachlan M J (2009). Chem Soc Rev.

[3] Li P-F, Chen C-F (2012). J Org Chem.

[4] Chen C-F (2011). Chem Commun.

[5] Jiang Y, Chen C-F (2011). Eur J Org Chem.

[6] Chen C-F, Ma Y-X (2013). Iptycene Chemistry: from Synthesis to Applications.

[7] Swager T M (2008). Acc Chem Res.

[8] VanVeller B, Schipper D J, Swager T M (2012). J Am Chem Soc.

[9] Bräse S, Gil C, Knepper K, Zimmermann V (2005). Angew Chem, Int Ed.

[10] Driver T G (2010). Org Biomol Chem.

[11] Beckmann H S G, Wittmann V, Bräse S, Banert K (2010). Azides in carbohydrate chemistry. Organic Azides.

[12] Lahann J, Lahann J (2009). Click Chemistry for Biotechnology and Materials Science.

[13] Astruc D, Liang L, Rapakousiou A, Ruiz J (2012). Acc Chem Res.

[14] Golas P L, Matyjaszewski K (2010). Chem Soc Rev.

[15] Finn M G, Fokin V V (2010). Chem Soc Rev.

[16] Sekhon B S (2012). J Pharm Educ Res.

[17] He X-P, Xie J, Tang Y, Li J, Chen G-R (2012). Curr Med Chem.

[18] Beal D M, Jones L H (2012). Angew Chem, Int Ed.

[19] Zhang X, Zhang Y (2013). Molecules.

[20] Berenguer J R, Bernechea M, Forniés J, Lalinde E, Torroba J (2005). Organometallics.

[21] Vicente J, Chicote M-T, Alvarez-Falcón M M, Jones P G (2005). Organometallics.

[22] Hua Y, Flood A H (2010). Chem Soc Rev.

[23] Wong C-H, Zimmerman S C (2013). Chem Commun.

[24] Gao C, Yan D (2004). Prog Polym Sci.

[25] Fahrenbach A C, Stoddart J F (2011). Chem–Asian J.

[26] Straub T, Boesenberg C, Gekeler V, Boege F (1997). Biochemistry.

[27] Hu Z, Kong F, Si M, Tian K, Yu L X, Young C Y, Lou H (2013). PLoS One.

[28] Gurova K (2009). Future Oncol.

[29] Perchellet E M, Magill M J, Huang X, Brantis C E, Hua D H, Perchellet J-P (1999). Anti-Cancer Drugs.

[30] Wang B, Perchellet E M, Wang Y, Tamura M, Hua D H, Perchellet J-P H (2003). Anti-Cancer Drugs.

[31] Perchellet E M, Wang Y, Lou K, Zhao H, Battina S K, Hua D H, Perchellet J-P (2007). Anticancer Res.

[32] Perchellet E M, Sperfslage B J, Wang Y, Huang X, Tamura M, Hua D H, Perchellet J-P (2002). Anti-Cancer Drugs.

[33] Perchellet E M, Wang Y, Webe R L, Lou K, Hua D H, Perchellet J-P H (2004). Anti-Cancer Drugs.

[34] Wang Y, Perchellet E M, Tamura M, Hua D H, Perchellet J-P (2002). Cancer Lett.

[35] Kumari R, Bhowmick S, Das N, Das P (2013).

[36] Zhang C, Chen C-F (2006). J Org Chem.

[37] Furukawa H, Cordova K E, O’Keeffe M, Yaghi O M (2013). Science.

[38] Valvekens P, Vermoortele F, De Vos D (2013). Catal Sci Technol.

[39] Horike S, Umeyama D, Kitagawa S (2013). Acc Chem Res.

[40] Janiak C (2013). Chem Commun.

[41] Nakagaki S, Ferreira G K B, Ucoski G M, Dias de Freitas Castro K A (2013). Molecules.

[42] Wang C, Liu D, Lin W (2013). J Am Chem Soc.

[43] Kumar S M, Dhahagani K, Rajesh J, Nehru K, Annaraj J, Chakkaravarthi G, Rajagopal G (2013). Polyhedron.

[44] Dwyer T J, Geierstanger B H, Mrksich M, Dervan P B, Wemmer D E (1993). J Am Chem Soc.

[45] Pandya P, Islam M M, Kumar G S, Jayaram B, Kumar S (2010). J Chem Sci.

[46] Galyuk E N, Fridman A S, Vorobev V I, Haroutiunian S G, Sargsyan S A, Hauruk M M, Lando D Y (2008). J Biomol Struct Dyn.

[47] Gonzáez V M, Pérez J, Alonso C (1997). J Inorg Biochem.

[48] Malina J, Hannon M J, Brabec V (2008). Nucleic Acids Res.

[49] Arjmand F, Jamsheera A (2011). Spectrochim Acta, Part A.

[50] Sirajuddin M, Ali S, Badshah A (2013). J Photochem Photobiol, B.

