# Supplementary material for: Synthesis, characterization and DNA interaction studies of new triptycene derivatives
Source: Beilstein J Org Chem. 2014 Jun 5;10:1290–8. doi: 10.3762/bjoc.10.130 (PMC4077457; doi:10.3762/bjoc.10.130)
Supplement: File 1 — Synthesis and characterization of compounds 1–8. [file Beilstein_J_Org_Chem-10-1290-s001.pdf]

Synthesis, characterization and DNA interaction studies of new triptycene derivatives

Sourav Chakraborty, Snehasish Mondal, Rina Kumari, Sourav Bhowmick, Prolay Das and Neeladri Das*

Address: Department of Chemistry, Indian Institute of Technology Patna, Patna 800 013, Bihar, India

Email: Neeladri Das* - neeladri@iitp.ac.in

*Corresponding author

Synthesis and characterization of compounds 1–8

Table of contents

1. Experimental section: preparation of compounds **1**–**8**……...…………………......S2–S4
2. ^{1}H and ^{13}C NMR spectra of compound **1**.. S5
3. ^{1}H and ^{13}C NMR spectra of compound **2**.. S6
4. ^{1}H and ^{13}C NMR spectra of compound **3**.. S7
5. ^{1}H and ^{13}C NMR spectra of compound **4**.. S8
6. ^{1}H and ^{13}C NMR spectra of compound **5**.. S9
7. ^{1}H and ^{13}C NMR spectra of compound **6**...S10
8. ^{1}H and ^{31}P NMR spectra of compound **7**...S11
9. ^{1}H and ^{31}P NMR spectra of compound **8**...S12
10. LRMS data of compound **1** and **2**... S13
11. LRMS data of compound **3** and **4**... S14
12. LRMS data of compound **5** and **6**... S15
13. LRMS data of compound **7** and **8**... S16
14. T_m studies of ctDNA with compound **5** and **6**………..…………………………….S17
15. Fluorescence and UV–vis data for interaction of ctDNA with **1**–**8** ………… S17–S19
16. Emission and excitation spectra of compounds **1** and **2**…………………………..S20

Experimental section:

Preparation of compounds 1 and 2: To a solution of appropriate tribromotriptycene (100 mg, 0.203 mmol), $Pd(PPh_3)_4$ (49.2 mg, 0.04 mmol) and CuI (7.6 mg, 0.04 mmol) in freshly distilled triethylamine (10 mL) was added ethynyltrimethylsilane (119 μL, 0.73 mmol) under nitrogen atmosphere. The reaction mixture was heated at 70 °C for 12 hour with constant stirring. Then reaction mixture was cooled and triethylamine was removed under reduced pressure in a rotary evaporator. Pentane was added to the crude product and filtered over celite. The filtrate was evaporated to obtain the silyl-protected product. This was dissolved in dry THF (10 mL) and Bu_4NF (230 mg, 0.73 mmol) solution in dry THF (5 mL) was added. The reaction mixture was stirred for 2 h. at room temperature. The solvent was evaporated under reduced pressure and the crude product was chromatographed on silica gel using 5% dichloromethane in hexane as eluent to obtain the desired triethynyltriptycene **1** or **2** as white solid.

Compound 1: yield 54 mg, 81%; mp 174-176 °C. ^1H NMR (400 MHz, $CDCl_3$): δ 2.90 (s, 3H, ≡CH), 5.27 (s, 1H, -CH), 5.29 (s, 1H, -CH), 7.06-7.12 (m, 3H, Ar-H), 7.25 (d, J = 7.2 Hz, 3H, Ar-H), 7.43 (d, J = 9.2 Hz, 3H, Ar-H). ^{13}C NMR (75 MHz, $CDCl_3$): δ 52.7, 53.1, 77.1, 83.4, 119.1, 119.2, 127.2, 127.4, 128.5, 128.9, 143.1, 143.2, 146.3, 146.4. Anal. Calcd. for $C_{26}H_{14}$: C, 95.68; H, 4.32. Found: C, 95.72; H, 4.34. IR (KBr): 3266, 2960, 2916, 2848, 2101, 1466, 1261, 1191, 1095, 1029, 890 cm^{-1}. MS (EI): m/z 326.2.

Compound 2: yield 51 mg, 77%; mp 170-172 °C. ^1H NMR (500 MHz, $CDCl_3$): δ 2.97 (s, 3H, ≡C-H), 5.34 (s, 1H, -CH), 5.40 (s, 3H, -CH), 7.16-7.17 (m, 3H, Ar-H), 7.32 (d, J = 7.5 Hz, 3H, Ar-H), 7.49-7.5 (m, 3H, Ar-H). ^{13}C NMR (75 MHz, $CDCl_3$): δ 53.0, 53.5, 77.2, 83.5 119.3, 123.7, 127.3, 129.7, 144.3, 144.8. Anal. Calcd. for $C_{26}H_{14}$: C, 95.68; H, 4.32. Found: C, 95.71; H, 4.30. IR (KBr): 3261, 2963, 2918, 2850, 2105, 1462, 1265, 1196, 1100, 1025, 900 cm^{-1}. MS (EI): m/z 326.3.

Preparation of 3 and 4: To a stirred solution of triaminotriptycene (299 mg, 1 mmol) dissolved in 1:2 (v/v) ratio of HCl:H_2O solution (3 mL) cooled at 0–5 °C with an ice bath, sodium nitrite solution (215 mg, 3 mmol dissolved in 2 mL of ice-cold water) was added dropwise over 5 min. After 10 min of stirring, NaN_3 solution (203 mg, 3 mmol dissolved in 2 mL water) was added dropwise to obtained yellow precipitate. Temperature of the flask was gradually increased to room temperature. After stirring being stirred for additional 2 h, reaction mixture was extracted with dichloromethane, washed with water, and dried over anhydrous sodium sulfate. The solvent

was removed under reduced pressure and the crude product was chromatographed on silica gel with 1% dichloromethane in hexane as eluent to obtain the desired triazidotriptycene **3** or **4** as a white solid.

Compound 3: yield 306 mg, 81%; mp 84-86 °C. ^{1}H NMR (400 MHz, $CDCl_3$): δ 5.33 (s, 1H, -CH), 5.34 (s, 1H, -CH), 6.63-6.68 (m, 3H, Ar-H), 7.07 (s, 3H, Ar-H), 7.31-7.34 (m, 3H, Ar-H). ^{13}C NMR (125 MHz, $CDCl_3$): δ 52.8, 53.3, 114.9, 115.0, 115.8, 115.9, 124.8, 124.9, 137.4, 137.5, 141.0, 141.4, 146.5, 147.0. Anal. Calcd for $C_{20}H_{11}N_9$: C, 63.66; H, 2.94; N, 33.41. Found: C, 63.61; H, 2.90; N, 33.55. IR (KBr): 2959, 2924, 2853, 2108, 1601, 1470, 1299, 1191, 818 cm^{-1}. MS (EI): m/z 376.9.

Compound 4: yield 287 mg, 76%; mp 82-84 °C. ^{1}H NMR (400 MHz, $CDCl_3$): δ 5.35 (s, 1H, -CH), 5.36 (s, 1H, -CH), 6.68 (dd, J = 8, 2.4 Hz, 3H, Ar-H), 7.08-7.09 (m, 3H, Ar-H), 7.32 (d, J = 8 Hz, 3H, Ar-H). ^{13}C NMR (125 MHz, $CDCl_3$): δ 52.2, 53.8, 115.1, 115.9, 124.6, 137.2, 141.8, 146.0. Anal. Calcd for $C_{20}H_{11}N_9$: C, 63.66; H, 2.94; N, 33.41. Found: C, 63.70; H, 3.04; N, 33.58. IR (KBr): 2961, 2916, 2848, 2113, 1603, 1473, 1261, 1096, 803 cm^{-1}. MS (EI): m/z 377.0.

Preparation of 5 and 6: To a stirred solution of triethynyltriptycene (100 mg, 0.306 mmol) in dry THF under nitrogen atmosphere at −70 °C, n-BuLi (550 μl, 1.376 mmol, 2.5 M in hexane) was added dropwise and stirred for 1 h. CO_2 gas was then bubbled through the solution and the temperature of the flask was gradually increased from −70 °C to room temperature. The resulting mixture was quenched with water, washed twice with diethyl ether, and subsequently acidified with dilute hydrochloric acid to obtain the desired triptycenetripropiolic acid **5** or **6** as the sole isolated product (yellow solid).

Compound 5: yield 99 mg, 70%; mp >350 °C. ^{1}H NMR (300 MHz, DMSO-d6): δ 5.98 (s, 1H, -CH), 6.00 (s, 1H, -CH), 7.41-7.44 (m, 3H, Ar-H), 7.64 (d, J = 7.5 Hz, 3H, Ar-H), 7.79-7.80 (m, 3H, Ar-H). ^{13}C NMR (75 MHz, DMSO-d6): δ 51.6, 52.0, 80.7, 81.7, 83.7, 84.6, 115.5, 116.4, 124.2, 125.2, 127.3, 128.2, 130.1, 131.1, 144.0, 144.9, 146.1, 147.0, 153.7, 154.6. Anal. Calcd. for $C_{29}H_{14}O_6$: C, 75.98; H, 3.08. Found: C, 75.01; H, 3.12. IR (KBr): 2964, 2875, 2206, 1700, 1475, 1416, 1261, 1212, 1094, 1019, 801 cm^{-1}. MS (EI): m/z 458.4.

Compound 6: yield 96 mg, 68%; mp >350 °C. ^{1}H NMR (300 MHz, DMSO-d6): δ 5.85 (s, 1H, -CH), 5.93 (s, 1H, -CH), 7.32 (d, J = 7.5 Hz, 3H, Ar-H), 7.55 (d, J = 7.5 Hz, 3H, Ar-H), 7.67 (s, 3H, Ar-H). ^{13}C NMR (75 MHz, DMSO-d6): δ 50.6, 51.1, 79.3.7, 82.8, 118.0, 123.6, 126.4, 128.6, 144.5, 145.2, 153.3. Anal. Calcd. for $C_{29}H_{14}O_6$: C, 75.98; H, 3.08. Found: C, 75.21; H, 3.16. IR (KBr): 3291, 2965, 2210, 1710, 1471, 1301, 1275, 1213, 879, 794, 699 cm^{-1}. MS (EI): m/z 458.6.

Preparation of 7 and 8: To a stirred solution of triethynyltriptycene (100 mg, 0.306 mmol) in dry THF under nitrogen atmosphere at −70 °C, 2.5 M solution of *n*-BuLi in hexane (550 μL, 1.376 mmol) was added dropwise with stirring. The resulting suspension was warmed to 0 °C, then again cooled to −70 °C and treated with PPh_2Cl (303 mg, 1.373 mmol). The reaction mixture was allowed to reach room temperature and was stirred overnight. The solvent was evaporated under reduced pressure, the residue was washed with methanol (2*10 mL) and dried under vacuum. The crude product was dissolved in dichloromethane, treated with activated charcoal, and passed through a plug of silica gel. The pale yellow solution was evaporated and the resulting solid was further purified by column chromatography on silica gel with 30% ethyl acetate in hexane as eluent to obtain the desired tris(biphenylphosphino)ethynyltriptycene **7** or **8** as a white solid.

Compound 7: yield 191 mg, 71%; mp 114-116 °C. ^{1}H NMR (300 MHz, $CDCl_3$): δ 5.40 (s, 1H, -CH), 5.41 (s, 1H, -CH), 7.21-7.23 (m, 3H, Ar-H), 7.31-7.33 (m, 18H, Ar-H), 7.35-7.36 (m, 3H, Ar-H), 7.56 (broad, 3H, Ar-H), 7.59-7.64 (m, 12H, Ar-H). ^{31}P{^{1}H} NMR (202 MHz, $CDCl_3$): δ 8.92. Anal. Calcd. for $C_{62}H_{41}P_3$: C, 84.73; H, 4.70. Found: C, 84.91; H, 4.78. IR (KBr): 3052, 2927, 2855, 2152, 1584, 1466, 1434, 1183, 1095, 943, 774, 738, 692 cm^{-1}. MS (EI): m/z 879.7.

Compound 8: yield 177 mg, 66%; mp 111-113 °C. ^{1}H NMR (500 MHz, $CDCl_3$): δ 5.38 (s, 1H, -CH), 5.42 (s, 1H, -CH), 7.20-7.22 (m, 3H, Ar-H), 7.32-7.33 (m, 18H, Ar-H), 7.35 (broad, 3H, Ar-H), 7.53-7.55 (m, 3H, Ar-H), 7.60-7.63 (m, 12H, Ar-H). ^{31}P{^{1}H} NMR (202 MHz, $CDCl_3$): δ 9.05. Anal. Calcd. for $C_{62}H_{41}P_3$: C, 84.73; H, 4.70. Found: C, 84.86; H, 4.81. IR (KBr): 3048, 2923, 2855, 2144, 1586, 1466, 1430, 1179, 1093, 947, 853, 738, 692 cm^{-1}. MS (EI): m/z 879.6.

^1H NMR spectrum of **1**:

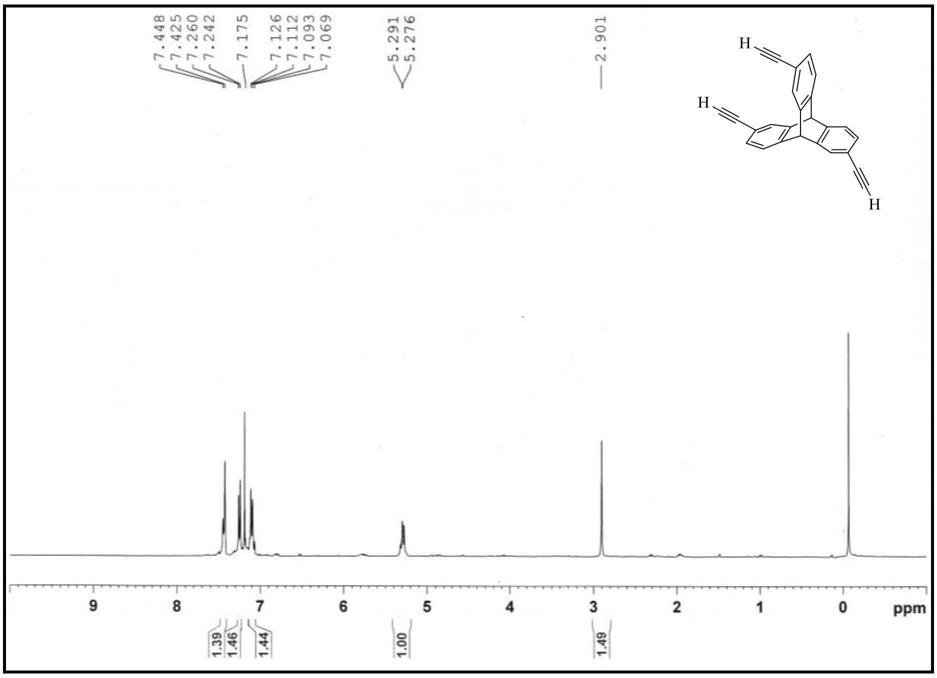

^{13}C NMR spectrum of **1**:

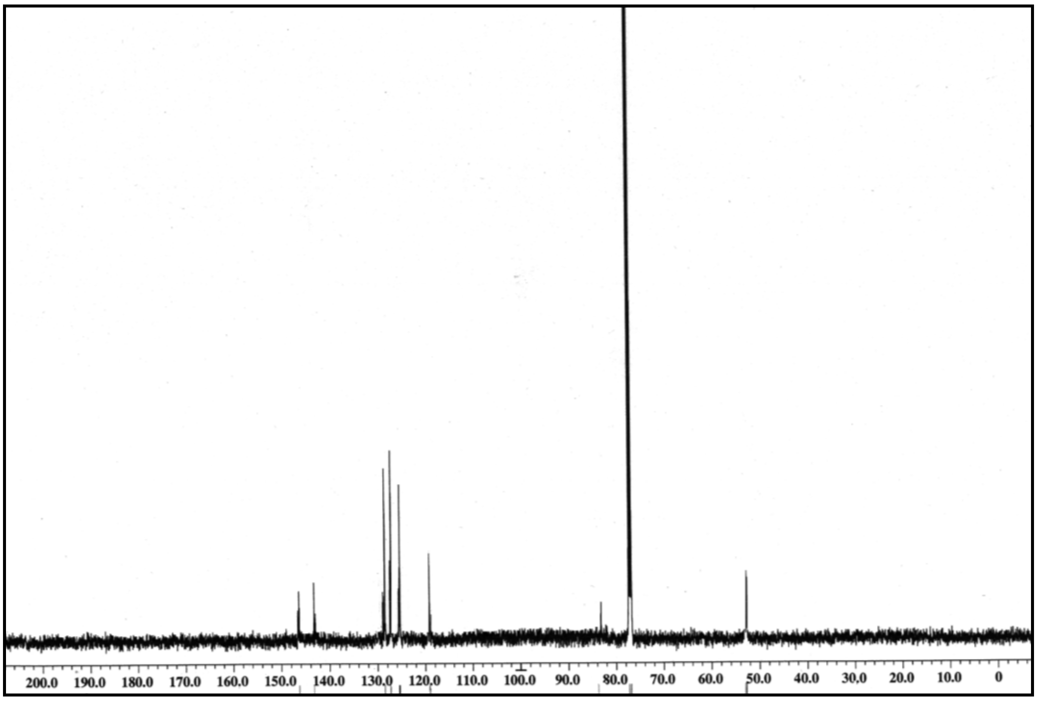

^{1}H NMR spectrum of **2**:

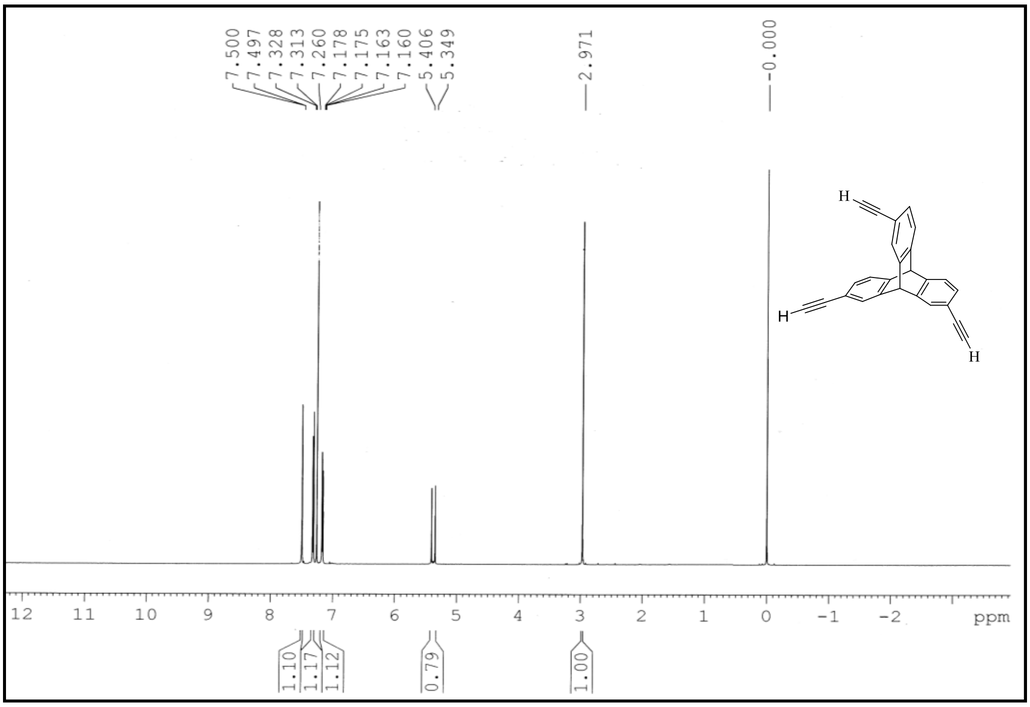

[13] C NMR spectrum of **2**:

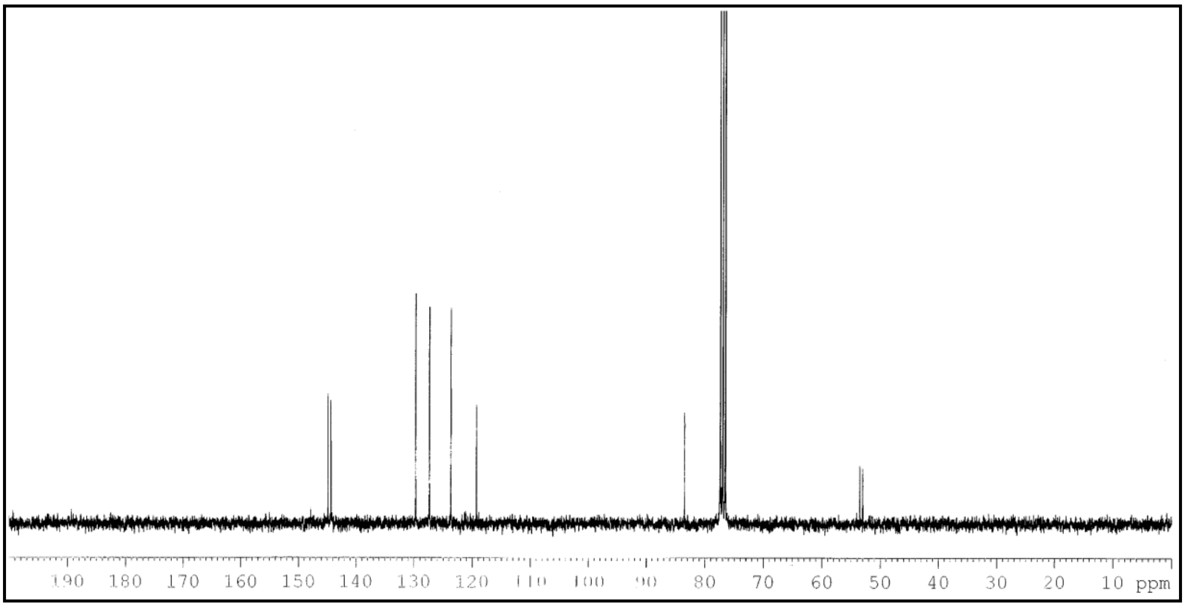

^{1}H NMR spectrum of **3:**

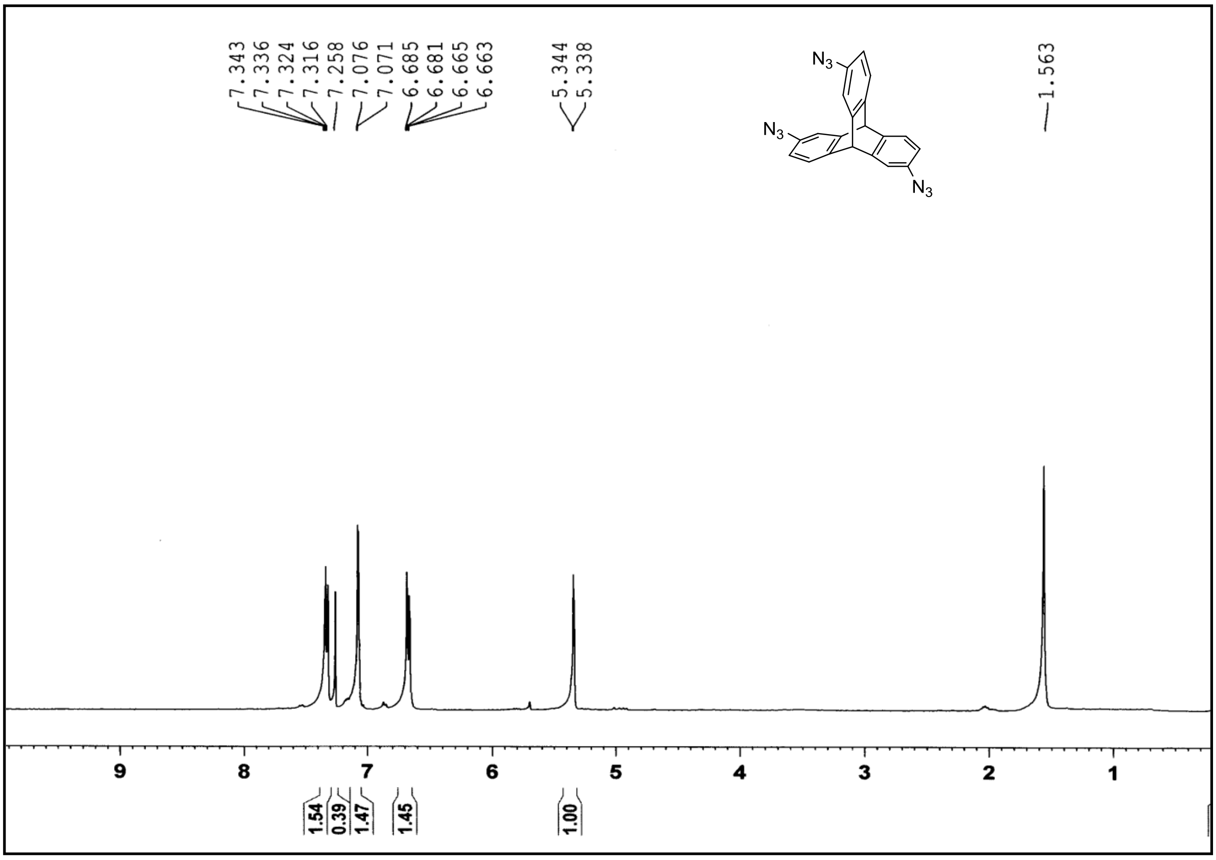

^{13}C NMR spectrum of **3:**

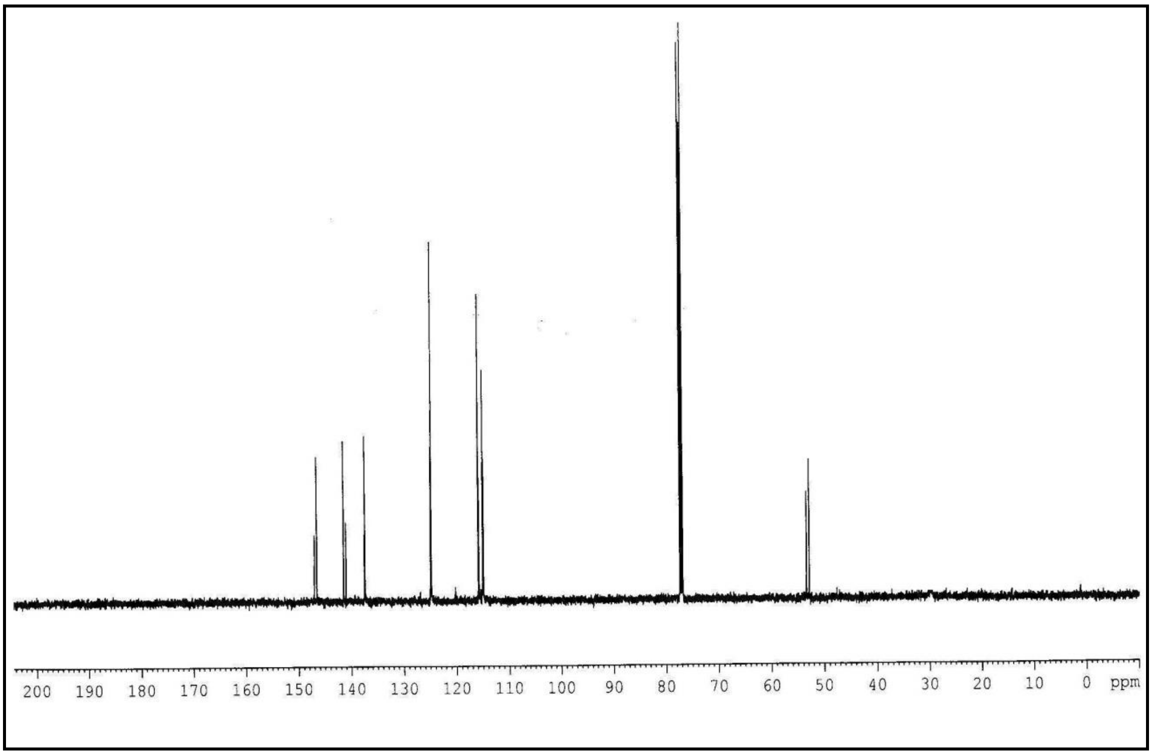

^{1}H NMR spectrum of **4:**

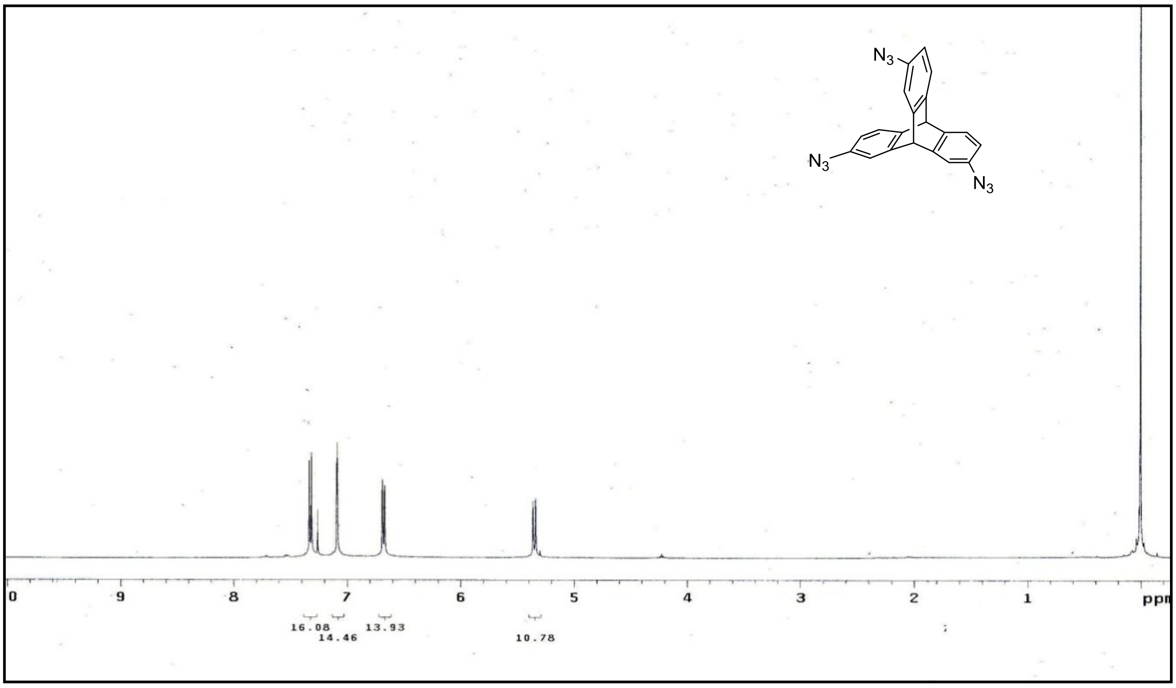

^{13}C NMR spectrum of **4:**

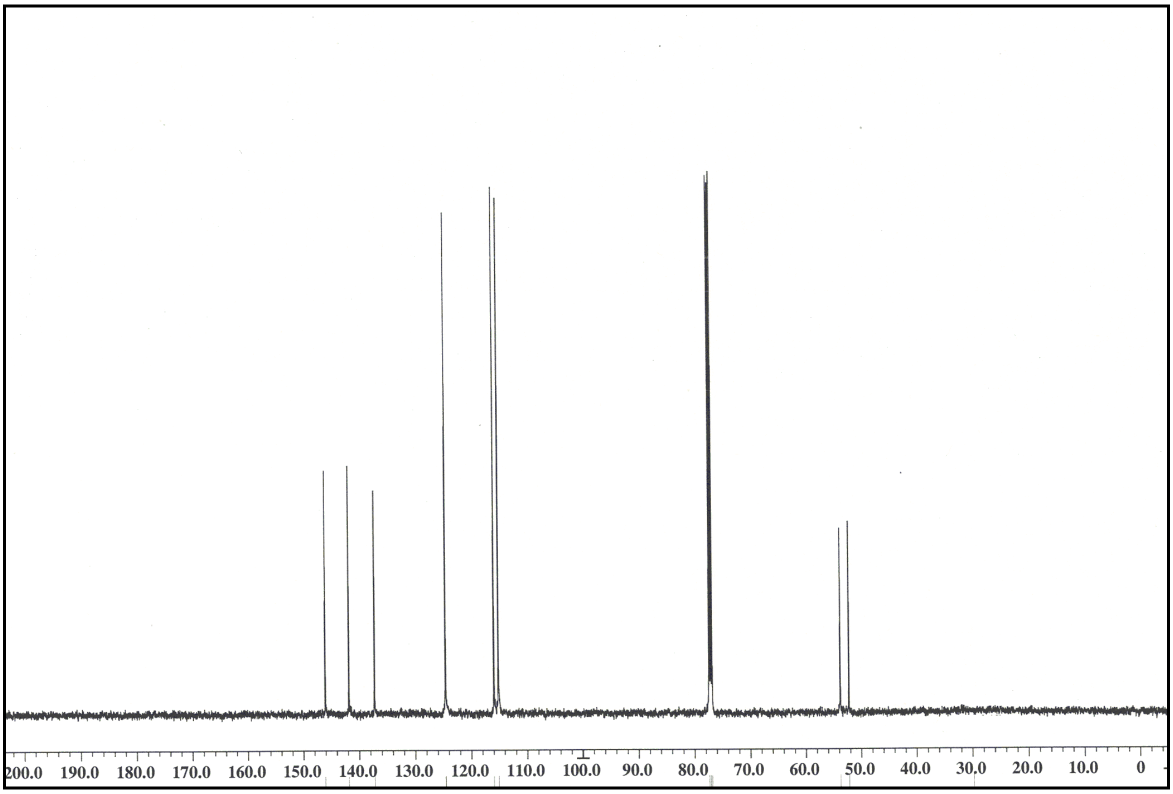

^{1}H NMR spectrum of **5:**

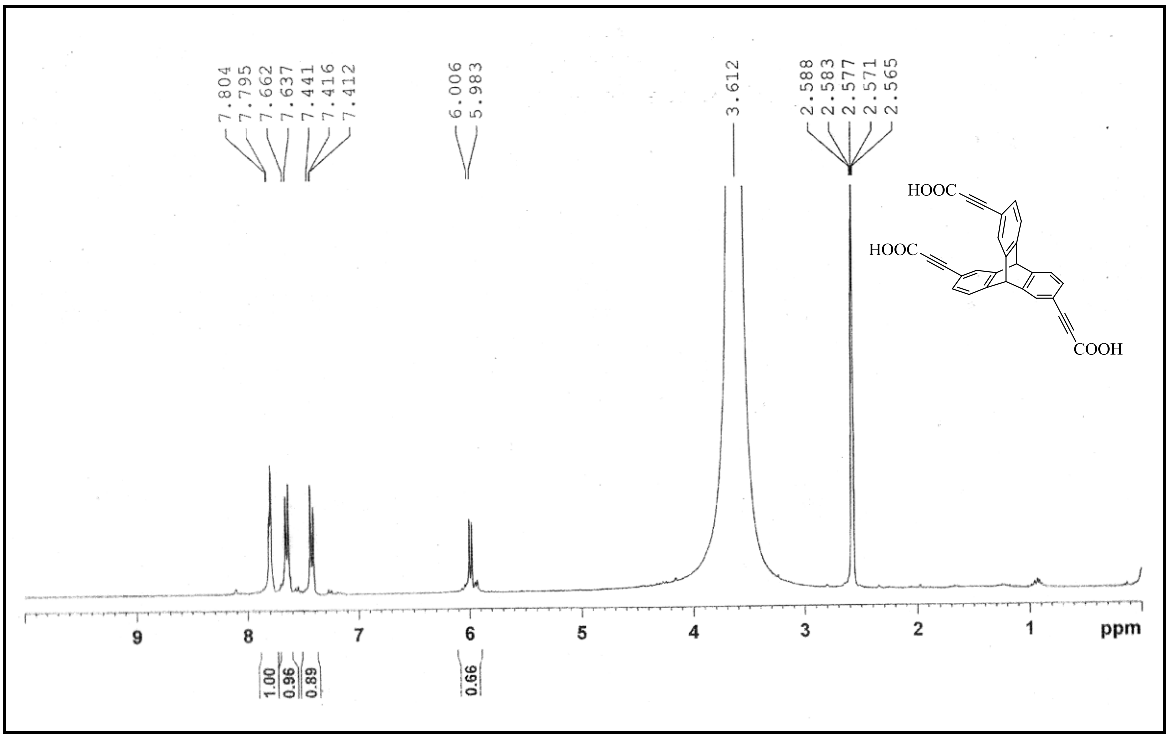

^{13}C NMR spectrum of **5:**

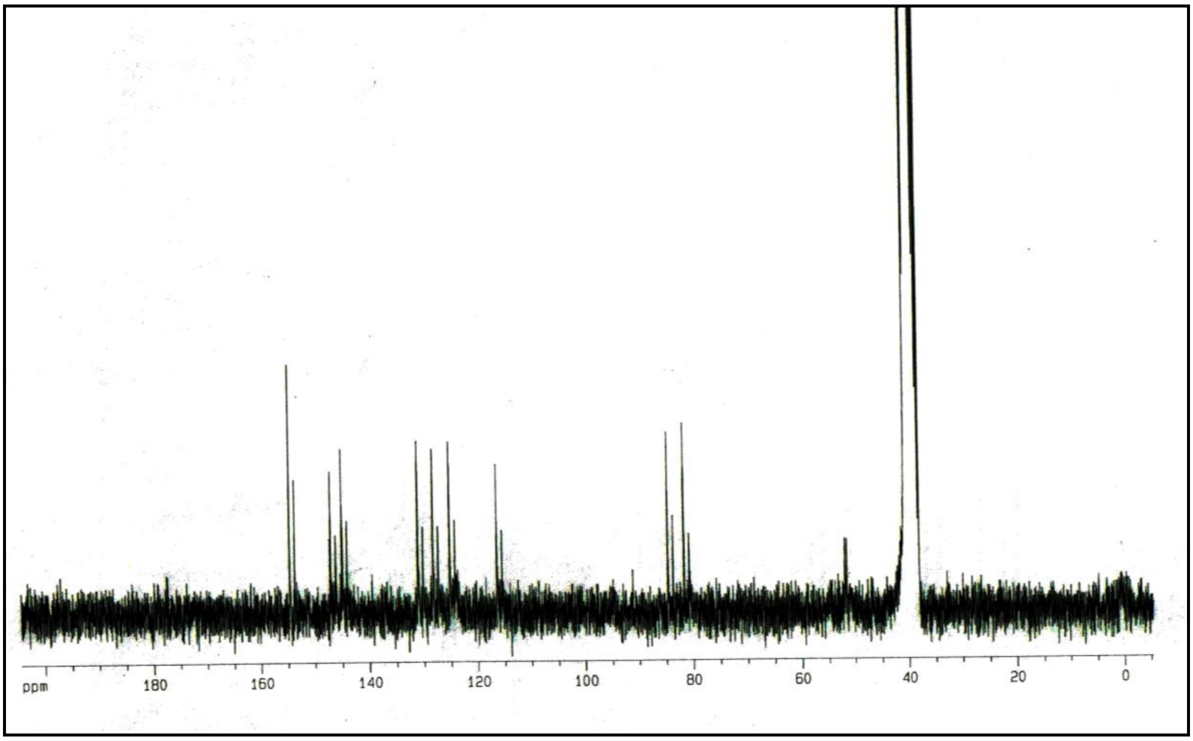

[1] H NMR spectrum of **6:**

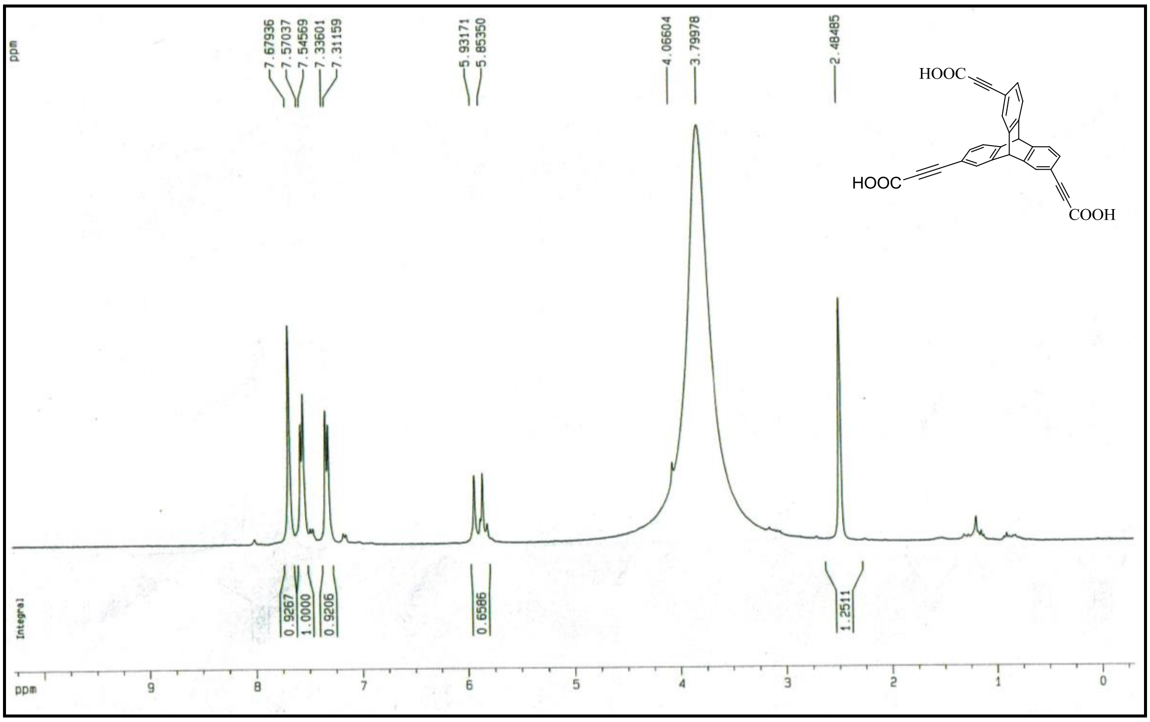

^{13}C NMR spectrum of **6:**

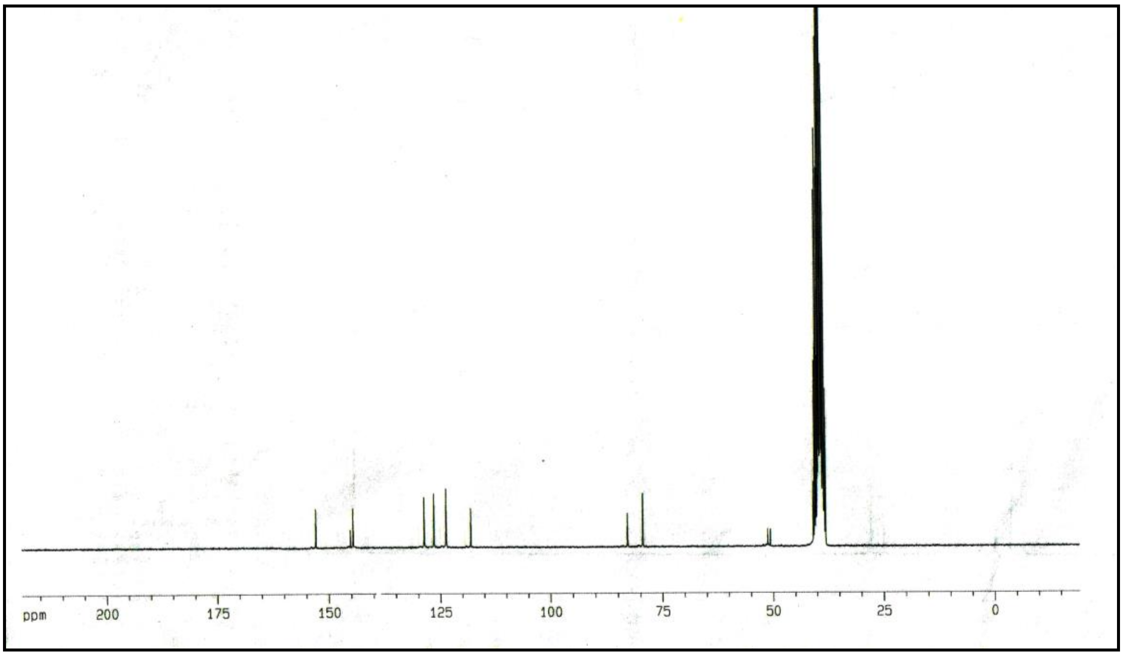

^{1}H NMR spectrum of **7:**

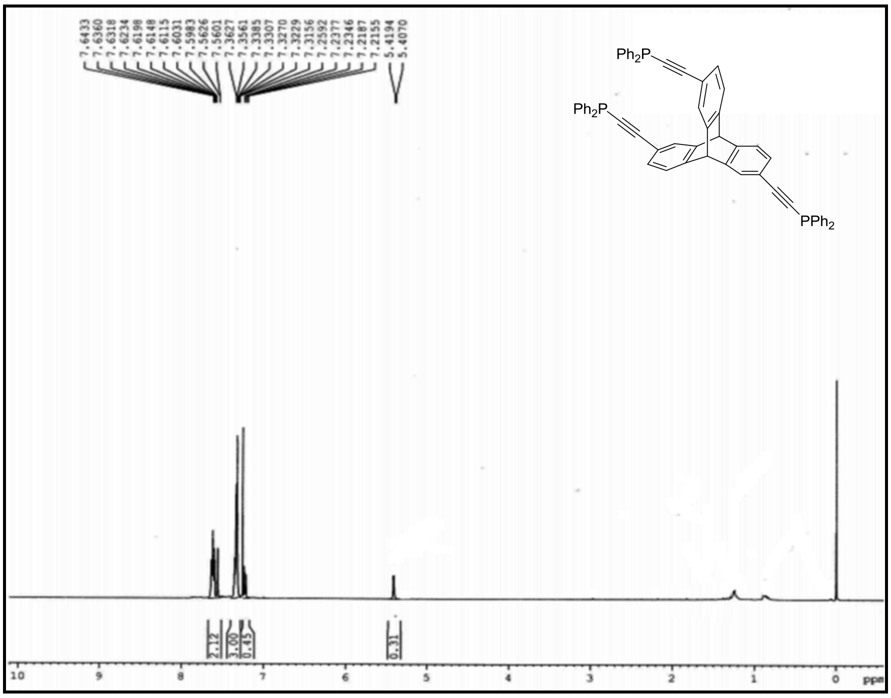

^{31}P NMR spectrum of **7:**

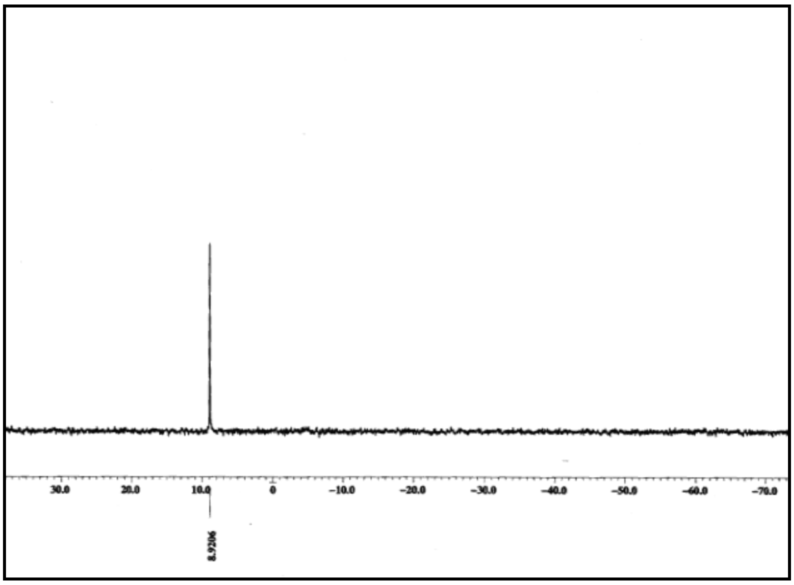

^{1}H NMR spectrum of **8:**

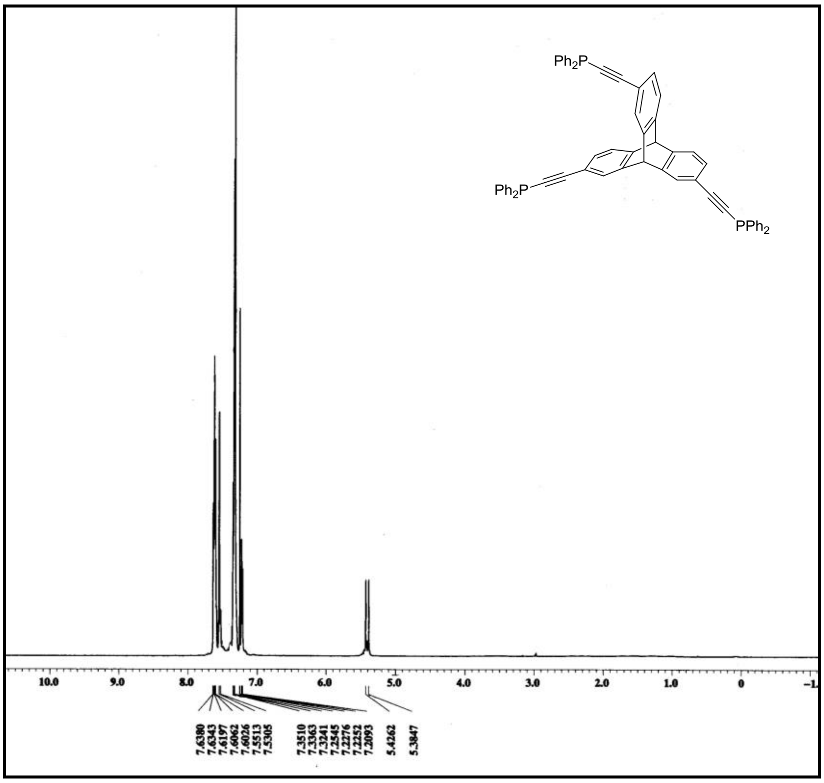

^{31}P NMR spectrum of **8:**

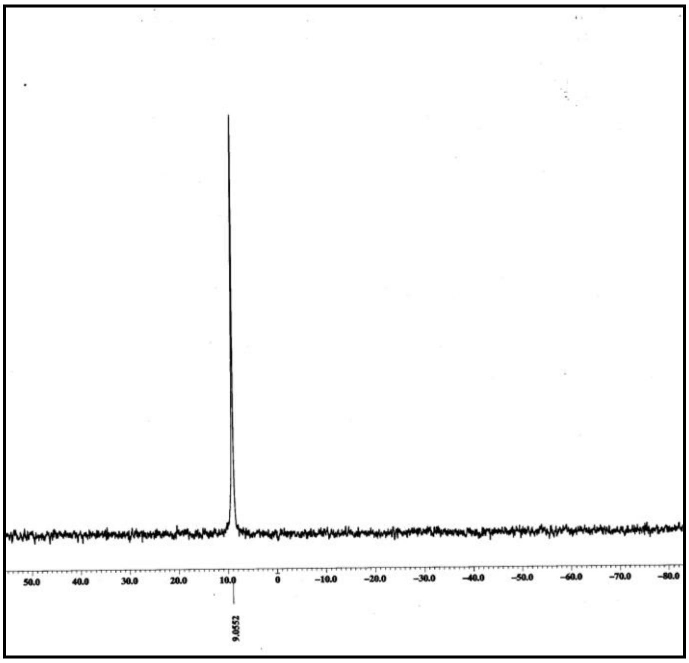

LRMS data of **1**:

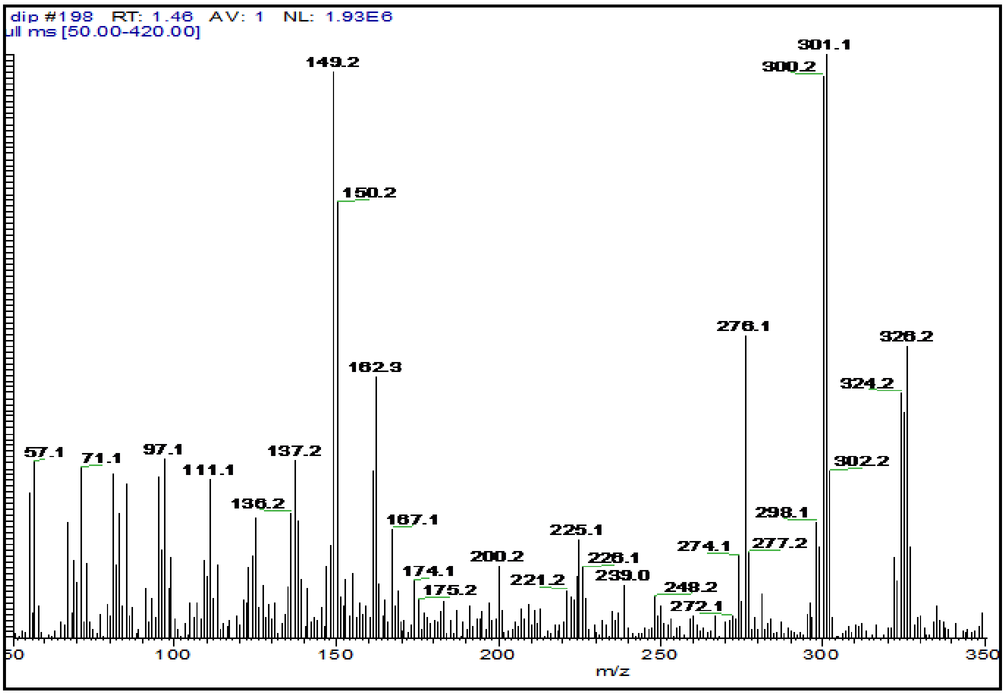

LRMS data of **2**:

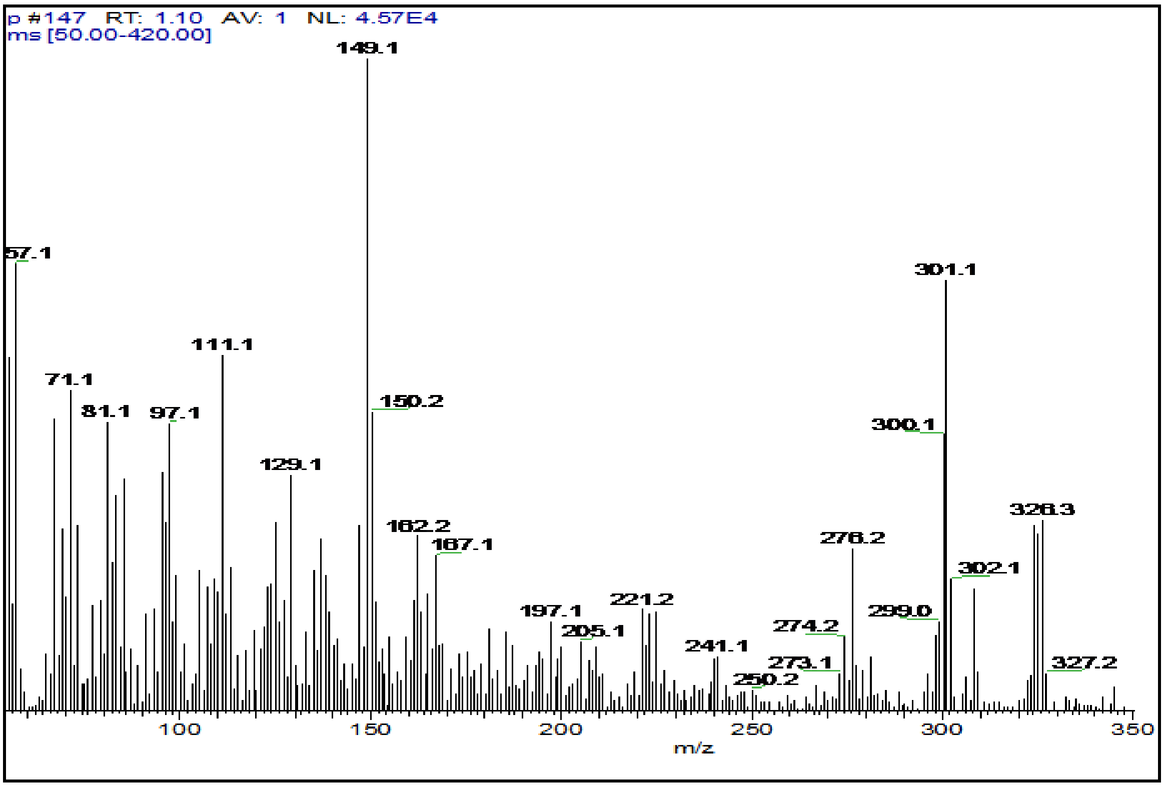

LR-MS dataof **3:**

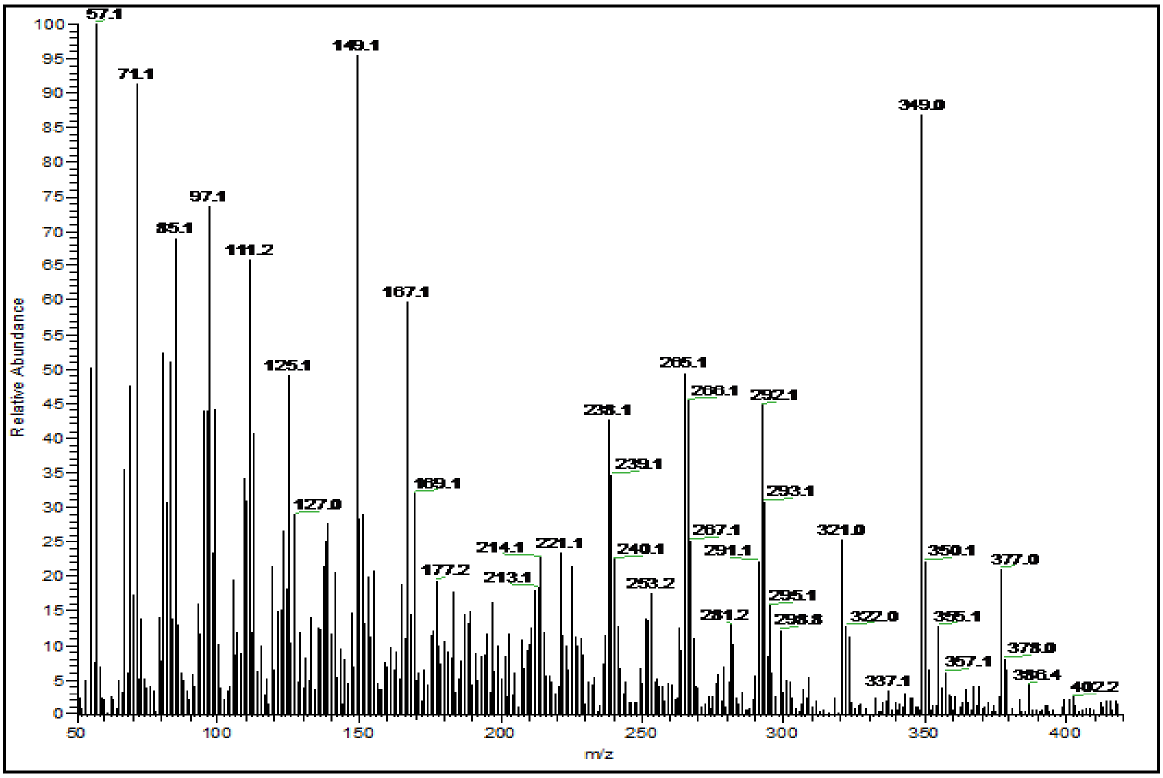

LR-MS data of **4:**

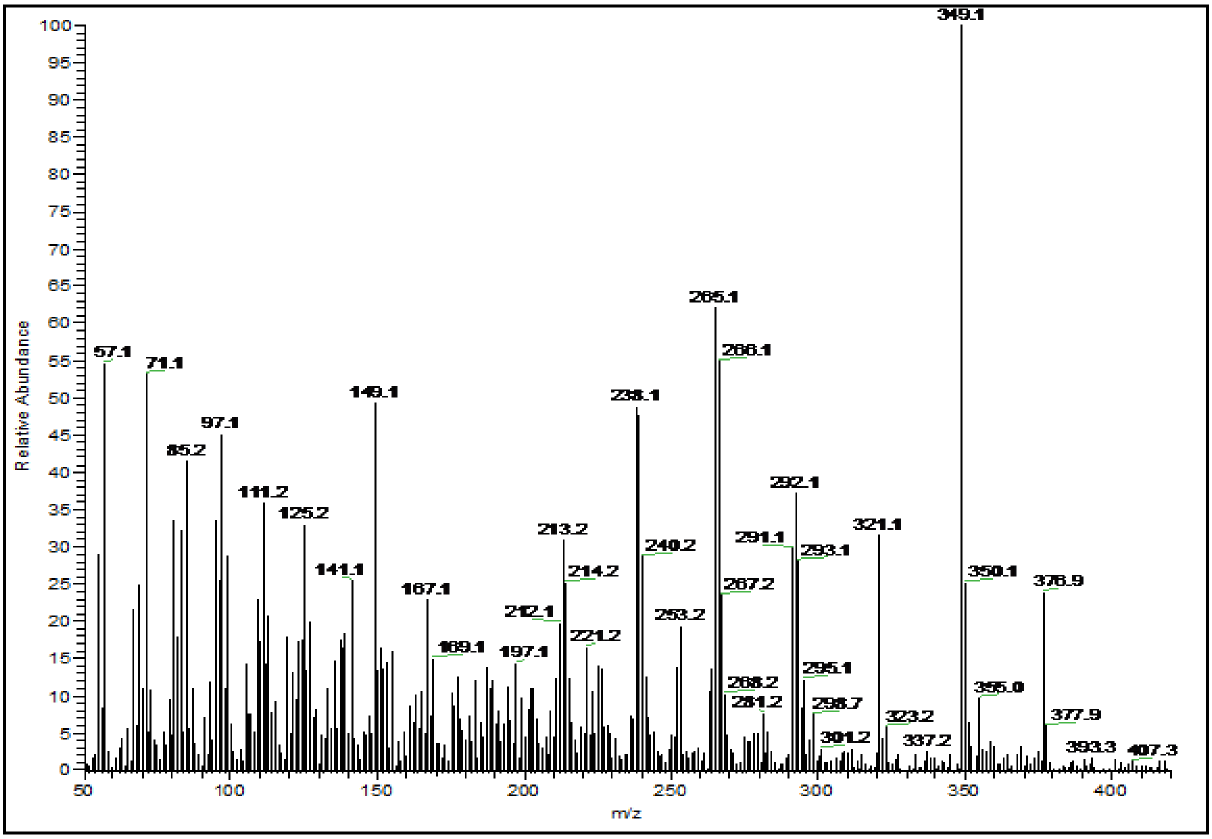

LRMS data of **5**:

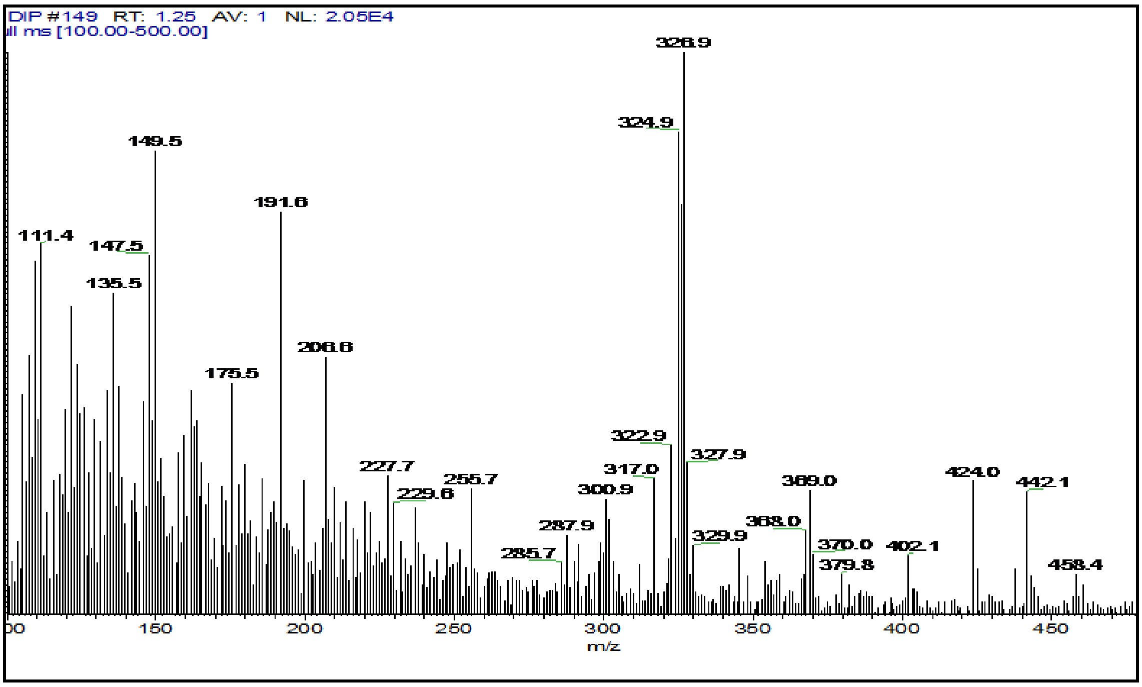

LRMS data of **6**:

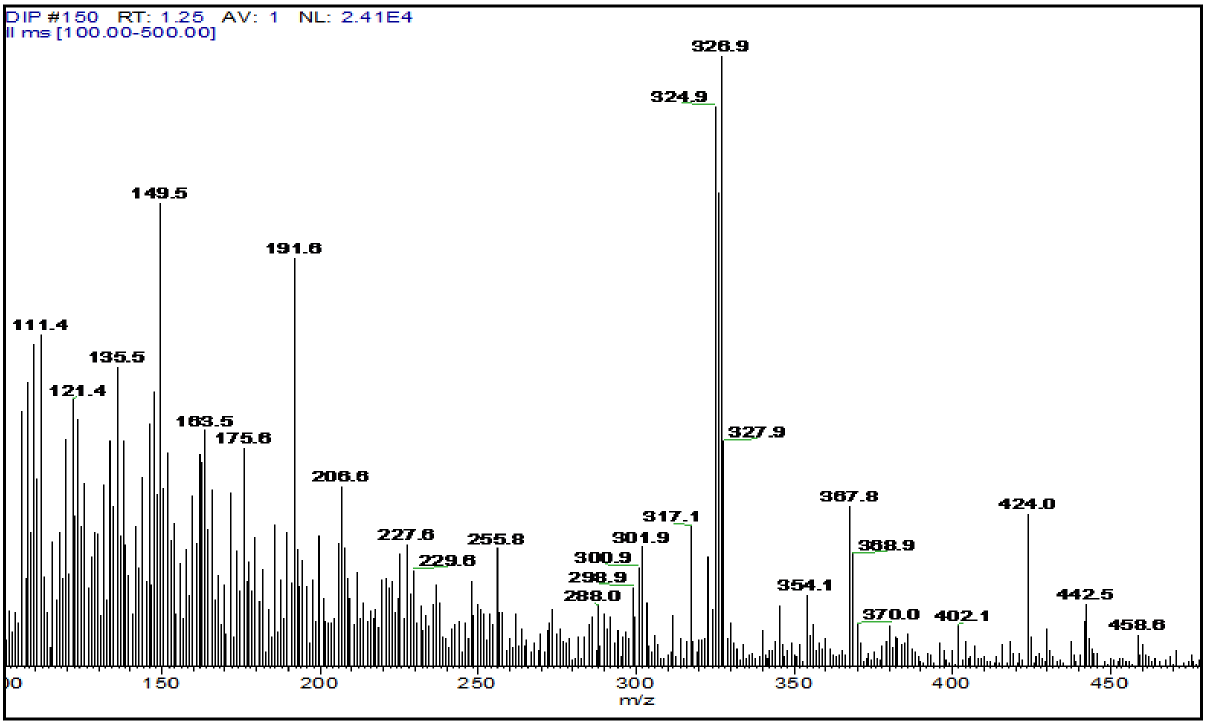

LRMS data of **7**:

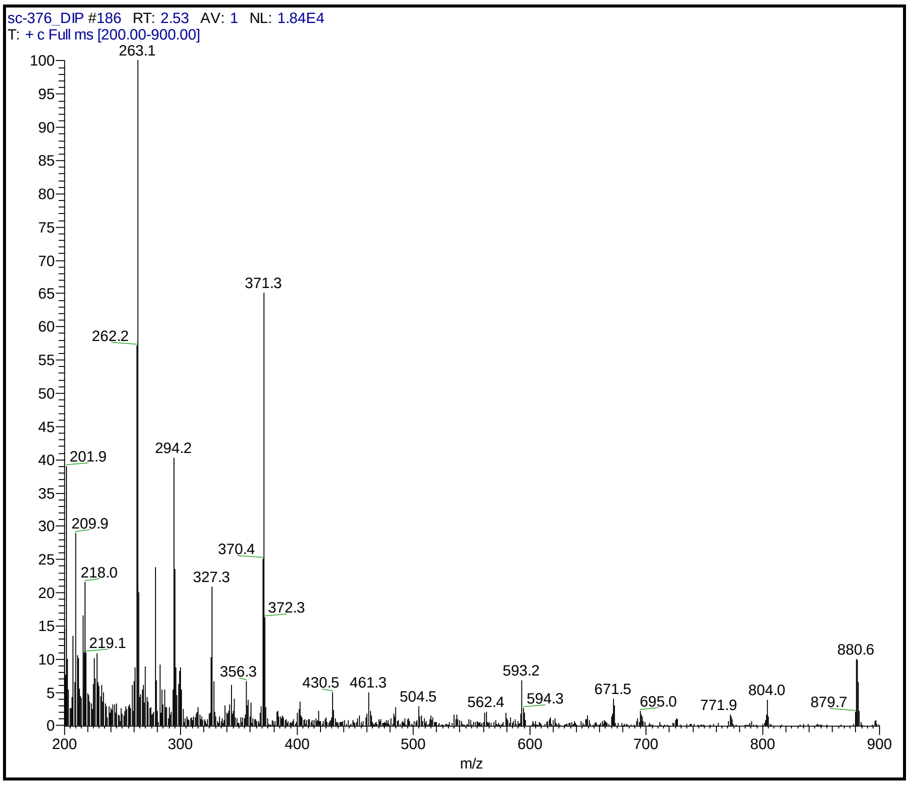

LRMS data of **8**:

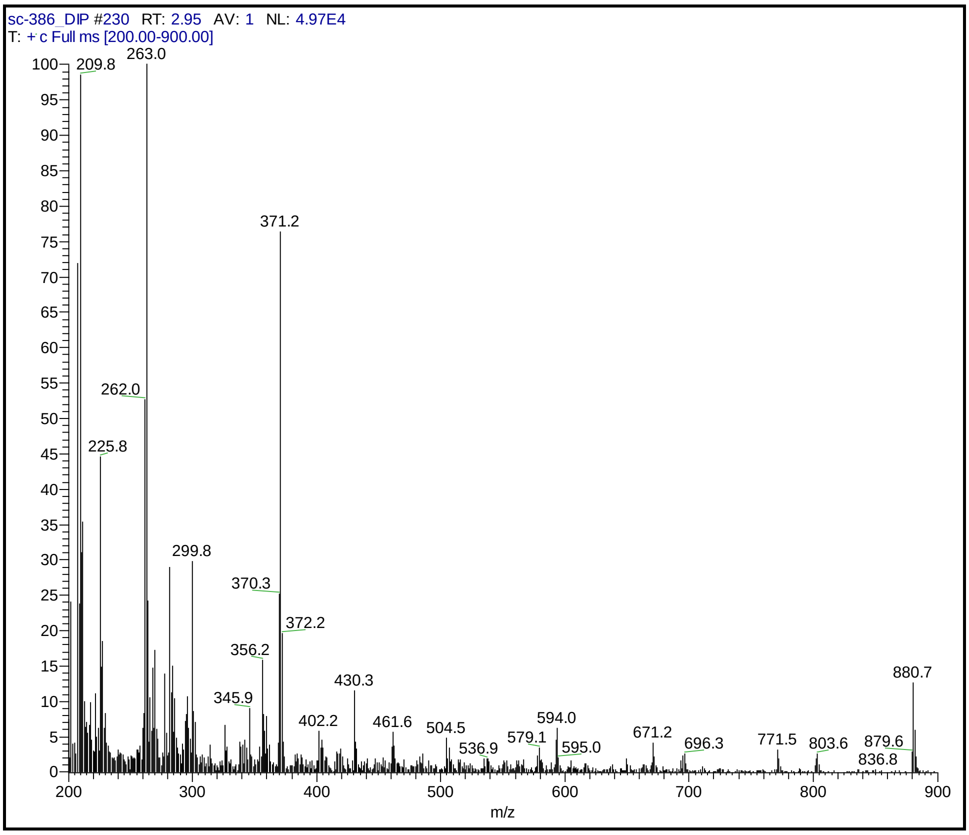

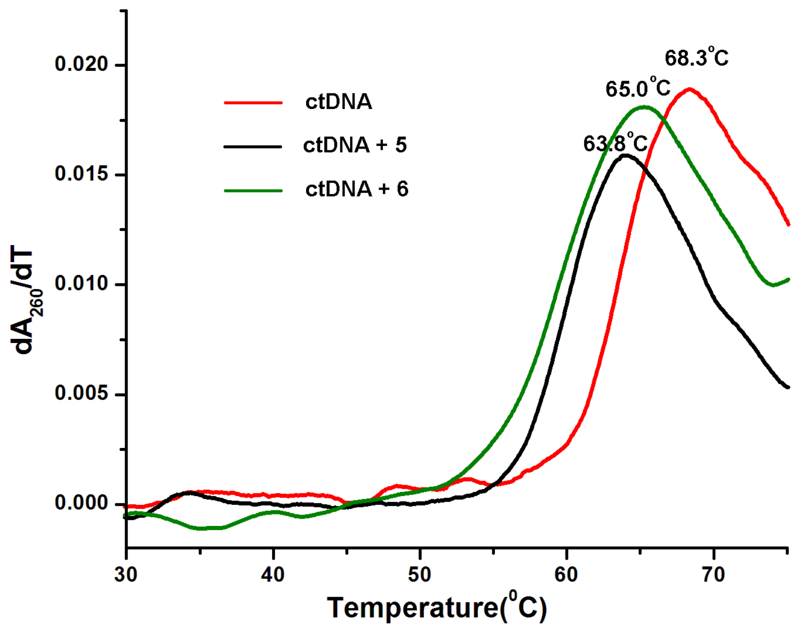

Figure S1: DNA melting temperature (T_m) studies with ctDNA in presence of **5** and **6**.

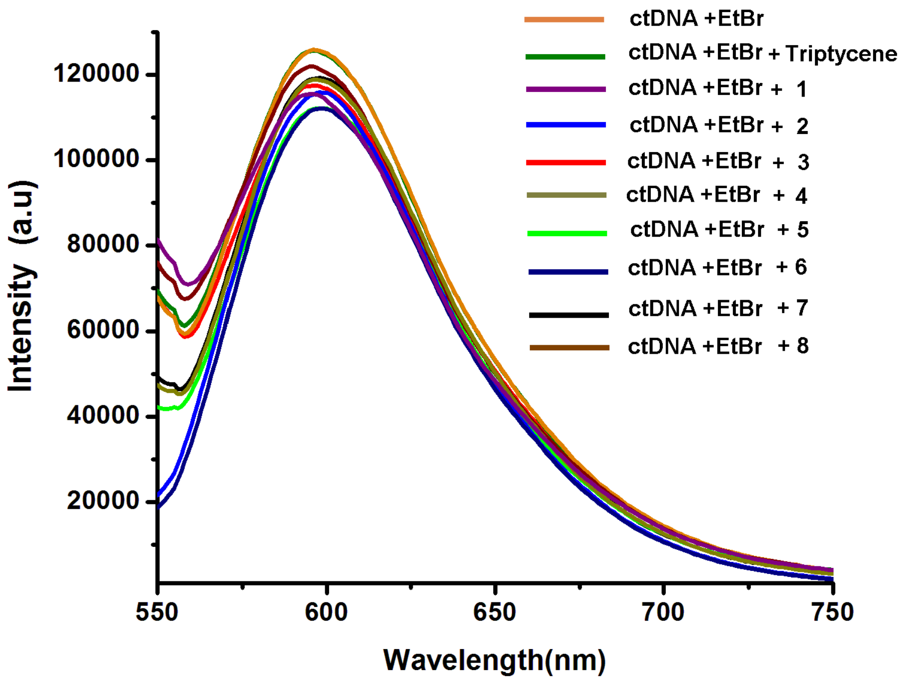

Figure S2: EtBr displacement assay for triptycene and compounds **1**–**8**.

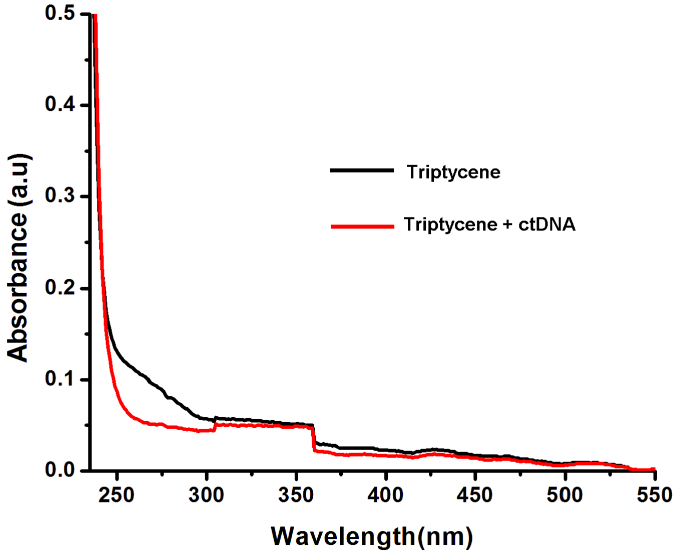

Figure S3: UV–vis spectra of triptycene in presence of ctDNA

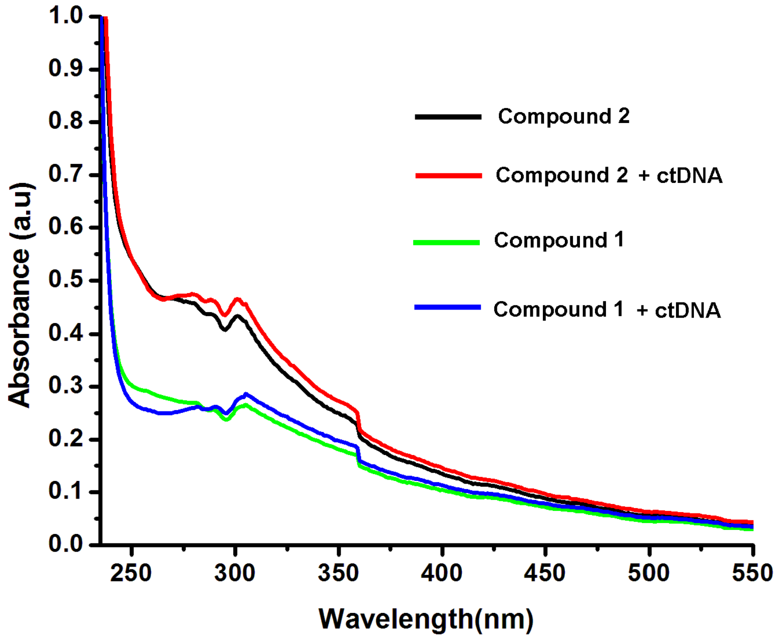

Figure S4: UV–vis spectra of compound **1** and **2** in presence of ctDNA

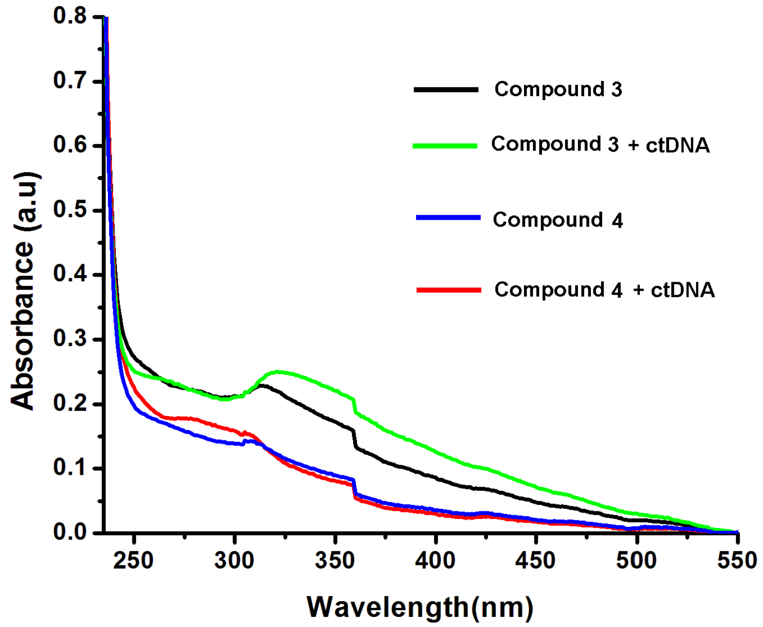

Figure S5: UV–vis spectra of compound **3** and **4** in presence of ctDNA

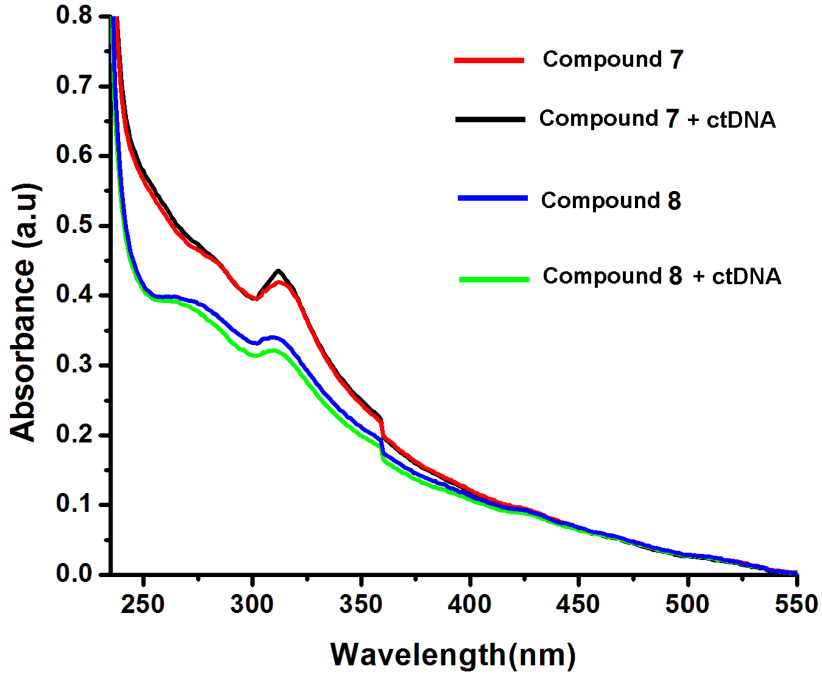

Figure S6: UV–vis spectra of compound **7** and **8** in presence of ctDNA

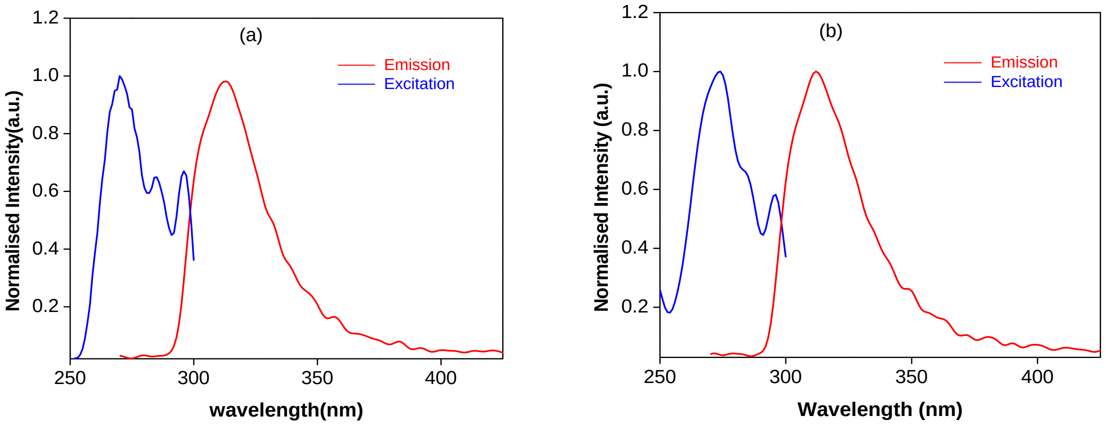

Figure S7: Normalized excitation and emission spectra of (a) TET **1** and (b) TET **2** in dmso solution at 298 K.
